# Oroxylin a Attenuates Limb Ischemia by Promoting Angiogenesis *via* Modulation of Endothelial Cell Migration

**DOI:** 10.3389/fphar.2021.705617

**Published:** 2021-07-30

**Authors:** Lusha Zhang, Lu Chen, Chunxiao Li, Hong Shi, Qianyi Wang, Wenjie Yang, Leyu Fang, Yuze Leng, Wei Sun, Mengyao Li, Yuejin Xue, Xiumei Gao, Hong Wang

**Affiliations:** ^1^ State Key Laboratory of Component-based Chinese Medicine, Tianjin University of Traditional Chinese Medicine, Tianjin, China; ^2^ Key Laboratory of Pharmacology of Traditional Chinese Medical Formula, Ministry of Education, Tianjin University of Traditional Chinese Medicine, Tianjin, China; ^3^ Tianjin Key Laboratory of Traditional Chinese Medicine Pharmacology, Tianjin, China; ^4^ Tianjin State Key Laboratory of Modern Chinese Medicine, Tianjin, China; ^5^ Institute of Traditional Chinese Medicine, Tianjin University of Traditional Chinese Medicine, Tianjin, China; ^6^ School of Integrative Medicine, Tianjin University of Traditional Chinese Medicine, Tianjin, China

**Keywords:** oroxylin A, hind limb ischemia, angiogenesis, endothelial cells, migration, inflammation

## Abstract

Oroxylin A (OA) has been shown to simultaneously increase coronary flow and provide a strong anti-inflammatory effect. In this study, we described the angiogenic properties of OA. OA treatment accelerated perfusion recovery, reduced tissue injury, and promoted angiogenesis after hindlimb ischemia (HLI). In addition, OA regulated the secretion of multiple cytokines, including vascular endothelial growth factor A (VEGFA), angiopoietin-2 (ANG-2), fibroblast growth factor-basic (FGF-2), and platelet derived growth factor BB (PDGF-BB). Specifically, those multiple cytokines were involved in cell migration, cell population proliferation, and angiogenesis. These effects were observed at 3, 7, and 14 days after HLI. In skeletal muscle cells, OA promoted the release of VEGFA and ANG-2. After OA treatment, the conditioned medium derived from skeletal muscle cells was found to significantly induce endothelial cell (EC) proliferation. OA also induced EC migration by activating the Ras homolog gene family member A (RhoA)/Rho-associated coiled-coil kinase 2 (ROCK-II) signaling pathway and the T-box20 (TBX20)/prokineticin 2 (PROK2) signaling pathway. In addition, OA was able to downregulate the number of macrophages and neutrophils, along with the secretion of interleukin-1β, at 3 days after HLI. These results expanded current knowledge about the beneficial effects of OA in angiogenesis and blood flow recovery. This research could open new directions for the development of novel therapeutic intervention for patients with peripheral artery disease (PAD).

## Introduction

Ischemia after artery obstruction that occurs during PAD is estimated to affect approximately 202 million adults worldwide (Gerhard-Herman et al., 2017; McDermott and Kibbe, 2017). The enhancement of angiogenesis and the resulting improvement of blood flow to the limb are the key restorative mechanisms in response to ischemia (Cooke and Losordo, 2015; Suzuki et al., 2016; Albrecht-Schgoer et al., 2017; Fan et al., 2018). At this point, treatments have not been fully effective at improving blood flow through angiogenesis in PAD (Suzuki et al., 2016; Gorenoi et al., 2017). Therefore, a better understanding of the underlying mechanisms involved in ischemic revascularization may help to optimize future clinical interventions.

Angiogenesis is a tightly regulated, multi-step process. When quiescent vessels sense angiogenic signals, such as guanosine triphosphatase (GTPase), vascular endothelial growth factor (VEGF), angiopoietin-2 (ANG-2), and fibroblast growth factor (FGFs), pericytes detach from the vessel wall and liberate themselves from the basement membrane *via* proteolytic degradation. Endothelial cells (ECs) loosen their junctions and the nascent vessel dilates (Carmeliet and Jain, 2011; Sun et al., 2017; Lee and Kang, 2018). In the early stage of angiogenesis, secretion of VEGF is closely related to inflammation, which can promote aggregation of neutrophils and production of inflammatory factor IL-1β (Saleh et al., 2015; Rajasagi et al., 2017). For therapeutic angiogenesis, it is not enough to identify pivotal regulators of angiogenesis as therapeutic targets to promote blood flow recovery. The surrounding inflammatory microenvironment should be considered at the same time to assess functional vasculature establishment in ischemic tissues (Liu et al., 2018; Minoshima et al., 2018). Previous research has shown that promoting angiogenesis while reducing inflammation can improve the prognosis of PAD (Zhang et al., 2016; Liu et al., 2018).

Oroxylin A (OA) (PubChem CID: 5320315) is one of the main bioactive compounds that is purified from the root of the medicinal herb *Scutellaria baicalensis* Georgi. This herb has been reported to have strong anti-inflammatory effects (Liu et al., 2012; Tseng et al., 2012; Wang et al., 2013). OA ameliorated some cardiac functions by increasing coronary flow (Liu et al., 2012; Tseng et al., 2016). These anti-inflammatory and pro-angiogenic effects of OA suggest that OA is most likely to establish functional vasculature in ischemic tissues and improve the prognosis of PAD. In this study, we investigated the therapeutic efficacy of OA for treating PAD and identified the molecular signaling pathways involved in the pro-angiogenic response both *in vivo* and *in vitro*.

## Materials and Methods

### Reagents

Avertin, simvastatin (Sim), collagenase II (C6885), and dispase II (D4693) were purchased from Sigma-Aldrich (MO, United States). Mouse XL Cytokine Array Kit (ARY028) and Mouse Angiogenesis Array Kit (ARY015) were purchased from R and D (MN, United States). α-SMA antibody (ab32575) was purchased from Abcam (Cambridge, United Kingdom). One Step TUNEL Apoptosis Assay Kit (C1089) was purchased from Beyotime Biotechnology (Shanghai, China). PE anti-F4/80 (565410), APC-Cy^TM^7 anti-Ly-6G (560600), PerCP anti-CD45 (561047) and matrigel (354234) were purchased from BD Company (NY, United States). Antibodies of VEGFA (ab51745), T-box20 (TBX20) (ab197386), and Prokineticin 2 (PROK2) (ab128293) were obtained from Abcam (Cambridge, United Kingdom). Antibodies for glyceraldehyde-3-phosphate dehydrogenase (GAPDH) (2118S), vascular endothelial growth factor receptor 2 (VEGFR2) (9698), Rho-associated coiled-coil kinase 2 (ROCK-II) (9029), Cofilin (5175), *p*-Cofilin (3313), β-actin (4970) were bought from Cell Signaling Technology (MA, United States). VEGFA (E-EL-M1292c), ANG-2 (E-EL-M0098c), PDGF-BB (E-EL-M0632c), IL-1β (E-EL-M0037c) mouse ELISA kits were obtained from Elabscience® (Wuhan, China). FGF basic mouse ELISA kit (MFB00) and VEGFA_165_ (293-VE-010) were obtained from R and D (MN, United States). Human umbilical vein endothelial cells (HUVECs, HUVEC-20001), endothelial cell growth medium (EGM) and fatal bovine serum (FBS) were obtained from Cyagen Biosciences Inc. (Guangzhou, China). Skeletal muscle cells (CP-H095) and skeletal muscle cells growth medium (CM-H095) were purchased from Procell Life Science & Technology Co., Ltd. (Wuhan, China). RhoA/Rac1/Cdc42 activation assay combo biochem kit™ (#BK030) was bought from Cytoskeleton (CO, United States). ROCK-II small interfering RNA (siRNA) (sc-29474), scrambled siRNA (sc-37007), and siRNA transfection reagent (sc-29528) were obtained from Santa Cruz Biotechnology (TX, United States).

### Animals

Male C57BL/6 mice of specific pathogen free, 22–25 g, were purchased from Beijing Weitong Lihua Experimental Animal Technology Co. Ltd. and maintained at the Animal Center of Institute of Biomedical Engineering, Chinese Academy of Medical Sciences (Tianjin, China). All animals were kept under 22–25°C and a 12 h light/dark cycle with standard food pellets and free access to tap water. All animal care and experimental procedures were approved by the Animal Ethics Committee of Tianjin University of Traditional Chinese Medicine and performed in accordance with the approved guidelines on the use of laboratory animals. The reference number of Institutional Animal Care and Use Committee (IACUC) is TCM-LAEC2019078.

### Surgical Hind Limb Ischemia Model

Mice were anesthetized with injection of Avertin (0.33 ml/20 g) into the abdominal cavity and maintained on a temperature-controlled water blanket at 37°C. Depilatory cream was applied to the limbs and the area was sterilized by 70% ethanol applications. A 5-mm vertical skin incision was made lateral to the abdomen and superficial to the inguinal ligament. The inguinal fat pad was separated from the peritoneal lining to reveal the proximal femoral artery branching from the internal iliac artery. The femoral artery and vein were then separated from the membrane sheath and two ligatures were tied around both vessels approximately 2-mm apart. Vessels were transected between the ligatures and the skin incision was closed with two discontinuous 6–0 silk sutures. Limb in sham mice were opened, dissected, and closed without vessel ligature and excision. After surgery the mice were sub-divided into four groups: 1. sham group (not HLI + saline); 2. saline group (HLI + saline); 3. OA group (HLI + OA, 10 mg/kg/d); 4. Sim group (HLI + Sim, 10 mg/kg/d). Treatments were given daily until euthanasia.

### Perfusion Imaging

The blood flow perfusion was measured by laser doppler perfusion imaging system both pre- and post-operatively and 3, 7, 14, 28 days after excision of the femoral artery in three groups. The blood flow images were collected by laser doppler high resolution imaging system (moor instruments, United States) and stored in the form of two-dimensional images of the whole area of the lower limbs. The ratio of blood flow in the ischemic (right) to the control (left) limb was calculated by moorLDI laser doppler imager review V 6.0 analysis software to show the recovery rate of ischemic hindlimb blood flow.

### Histological Analysis

The ischemic hindlimb muscle tissues were obtained 28 days after operation and fixed with 4% paraformaldehyde. The muscles were dehydrated, embedded, and transversely sectioned into 5 µm pieces. Gross examination of tissue injury was performed on hematoxylin and eosin (H and E) stained sections.

### Terminal Deoxynucleotidyl Transferase-Mediated dUTP Nick End-Labeling Assay

TUNEL staining was performed following the manufacturers’ instructions in paraffin sections. DAPI staining was used to count the total number of nuclei. Apoptotic myocytes were quantified and classified as TUNEL^+^ cells.

### Immunofluorescence

For immunofluorescent labeling, the mice were intravenously injected with 50 µL Griffonia (Bandeiraea) Simplicifolia Lectin I 30 min before sacrifices. The paraffin sections were incubated with Griffonia (Bandeiraea) Simplicifolia Lectin I and α-SMA antibodies overnight at 4°C. The following day, the tissue sections were incubated with DyLight^®^594 ANTI-GOAT IgG (H + L) and FITC mouse anti-rabbit (1:200) for 1 h in the dark at room temperature. All sections were counterstained with DAPI.

### Proteome Antibody Array Analysis

To determine the regulated proteins in the gastrocnemius muscle after ischemia, mice were euthanized after 3, 7, and 14 days. After adding Triton X-100 to a final concentration of 1%, tissue homogenates were frozen at −80°C for 2 h, then quickly thawed and centrifuged at 10,000 g for 5 min to remove cellular debris. The upper aqueous phase was transferred to a new tube for proteome antibody array analysis, which included Mouse XL Cytokine Array Kit for 3 days and Mouse Angiogenesis Array Kit for 7, 14 days after surgery.

### Flow Cytometry Analysis

The ischemic gastrocnemius muscles were digested in 1.8 ml Hanks Balanced Salt Solution with 7.2 mg collagenase II and 9 mg dispase II at 37°C for 1.5 h using GentleMACS™ Dissociator (MACS, German). The strained cells were stained with PE anti-F4/80, APC-Cy^TM^7 anti-Ly-6G, and PerCP anti-CD45. Single-cell suspensions were examined on a FACS Aria III flow cytometer.

### Western Blotting

For this experiment, frozen skeletal samples from hindlimbs were homogenized in lysis buffer. Protein concentration was measured by BCA method. Nine primary antibodies were used in the experiments: GAPDH, VEGFA, VEGFR2, ROCK2 (ROCK-II), TBX20, PROK2, Cofilin, *p*-Cofilin (Ser3), β-Actin. The reactive bands were developed using chemiluminescence according to the manufacturer’s instruction. A semi-quantitative estimation can be derived from the size and color intensity of the band on the blot membrane.

### Enzyme-Linked Immunosorbent Assay

Secreted VEGFA, ANG-2, FGF-2, PDGF-BB, and IL-1β in tissue lysates or serum were determined by ELISA according to the manufacturer’s instructions.

### Cell Culture

We obtained HUVECs from Cyagen Biosciences Inc. (HUVEC-20001) and skeletal muscle cells from Procell Life Science and Technology Co., Ltd. (CP-H095). Endothelial cell growth medium (EGM) contains endothelial cell basal medium (EBM), fetal bovine serum (10%), penicillin-streptomycin (1%), glutamine (1%), ECGS (1%), heparin (1%). EGM was used to culture HUVECs and skeletal muscle cells growth medium (CM-H095) was used to culture skeletal muscle cells in a cell incubator consisting of 5% humidified CO_2_ at 37°C.

### Preparation of Conditioned Medium

Skeletal muscle cells within a range of three passages were seeded in skeletal muscle cells growth medium in 6-well plate at a density of 1×10^4^ cells per well. HUVECs within a range of five passages were seeded in EGM in 96-well plate at a density of 3×10^3^ cells per well. When skeletal muscle cells density reached 60%, OA or DMSO (control) was given to intervene skeletal muscle cells for 24 h and then the medium of skeletal muscle cell was centrifuged at 1000 rpm for 5 min, and the supernatant was collected, aliquoted and stored in a refrigerator at −80°C (to detect secreted cell growth factors) or used for the culture of HUVECs. After OA or DMSO acted on skeletal muscle cells, the supernatants were the conditioned medium containing growth factors. The medium of HUVECs was removed and the skeletal muscle cells-conditioned medium were added to HUVECs for 24 h and then cell proliferation was measured using a BrdU kit.

### Tube Formation Assay

Chilled Matrigel (50 μL) was added to each well of a 96-well plate and allowed to polymerize for 30 min at 37°C. Cell suspension was added per well and incubated for 18 h. Brightfield images were taken using a Nikon microscope and quantified using Photoshop CS5 software (Adobe Systems, United States).

### Scratch Assays

With the use of WOUNDMAKER 96 (ESSEN BIOSCIENCE, United States), horizontal scratches were made across the culture dish. The scratches were imaged using IncuCyte Zoom (ESSEN, United States).

### Cell Proliferation Assays

Cell proliferation was measured using a BrdU kit (Roche, United States) and quantitated on a microplate reader, whereas viability and total cell number were determined in separate assays using an MTT assay kit (Invitrogen, United States).

### RNA Interference

For ROCK-II knock-down, HUVECs were transfected with ROCK-II siRNA (10 μM) or scrambled siRNA (10 μM) using siRNA transfection reagent according to the manufacturer’s instructions. Knockdown efficiency was assessed by quantitative reverse transcriptase polymerase chain reaction (qRT-PCR) 48 h later.

### Quantitative Reverse Transcriptase Polymerase Chain Reaction

qRT-PCR was performed in QuantStudio six Flex. The following human primers were used:

*GAPDH*:

Forward CAA​CGT​GTC​AGT​GGT​GGA​CCT​G.

Reverse GTG​TCG​CTG​TTG​AAG​TCA​GAG​GAG.

*VEGFR2*:

Forward CGG​ACA​GTG​GTA​TGG​TTC​TTG​CC.

Reverse GTG​GTG​TCT​GTG​TCA​TCG​GAG​TG.

*ROCK-II*:

Forward TCG​TCA​CAA​GGC​ATC​GCA​GAA​G.

Reverse CCA​CCA​GGC​ATG​TAC​TCC​ATT​ACC.

*TBX20*:

Forward ACC​AGC​ACA​GCA​TCC​ATA​GCA​AC.

Reverse GCA​ATG​GCC​GAT​GGT​GTC​AGA​G.

*PROK2*:

Forward ACT​CCT​GCT​CCT​CTT​GCT​GCT​G.

Reverse GCA​CAT​GCC​TCC​ACC​ACA​TTG​G.

### Transwell

A chemotactic migration assay was performed using transwell plates with 6.5 mm diameter polycarbonate filters (8 μm pore size). The upper and lower surfaces of the chamber were coated with fibronectin. EGM medium was placed in the lower wells. Cells were loaded into upper wells. The chamber was incubated at 37°C for 4 h. Migrated cells were stained with crystal violet and quantified using an optical microscope.

### Statistical Analysis

Statistical analysis was performed using SPSS software (version 17.0). Data were expressed as mean ± standard deviation (SD). Differences between two groups were estimated with Student’s *t*-test. Values of *p* < 0.05 were considered statistically significant.

## Results

### Oroxylin A Ameliorated Hindlimb Ischemia in Hind Limb Ischemia Mouse Model

**FIGURE 1:**
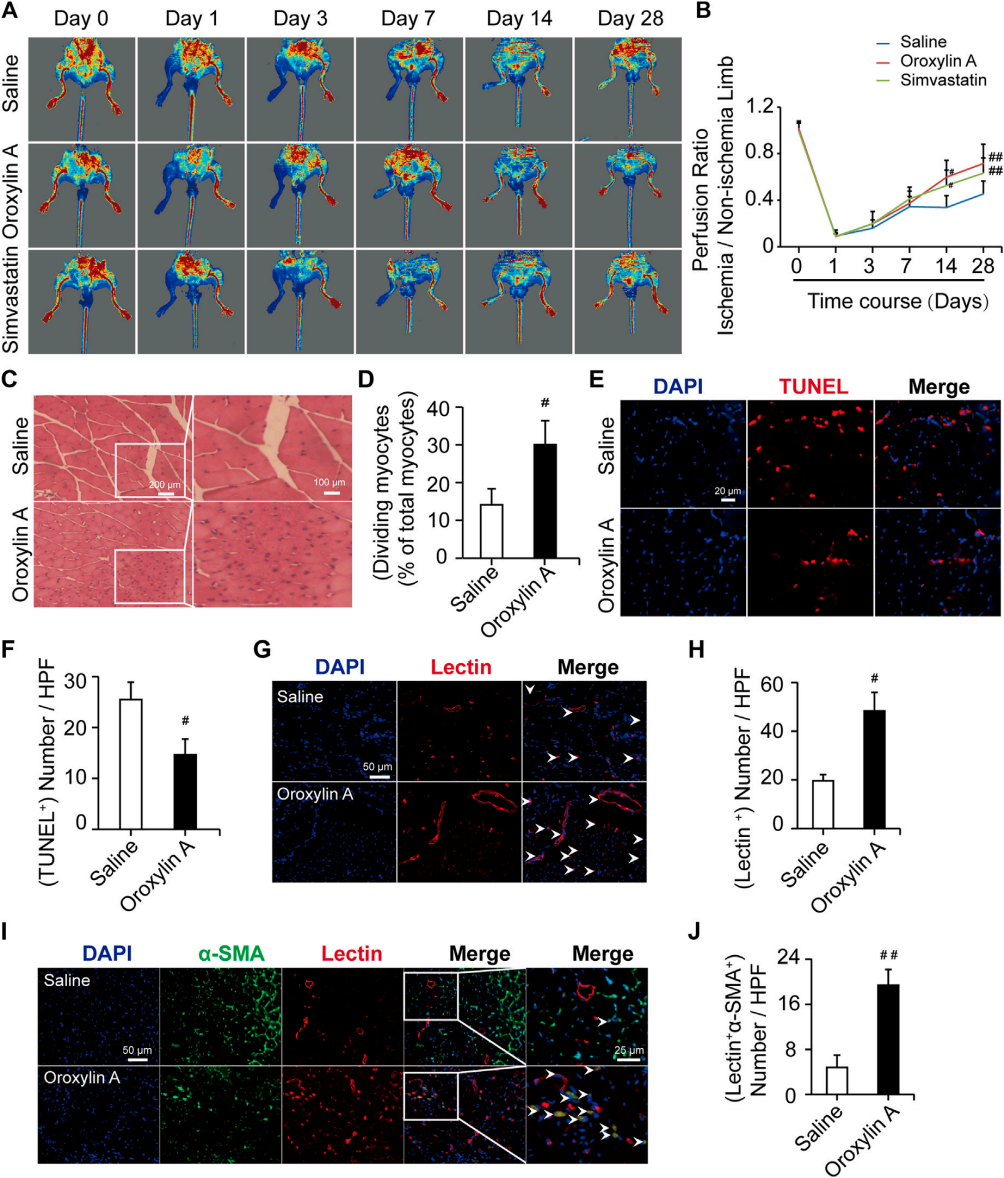
OA ameliorated hindlimb ischemia in a mouse model of HLI. **(A)** Typical blood flow perfusion photographs at different time (pre- and post-operatively, as well as 3, 7, 14, and 28 days after excision of the femoral artery) in three groups were presented. **(B)** Quantification of blood perfusion at different times was performed. Data are mean ± SD; *n* = 5–8/group. **(C,D)** H and E staining of muscle tissue in hindlimb confirmed the number of myocytes with centrally-located nuclei were increased by OA therapy. The scale bar represented 200 μm (left) and the scale bar represented 100 μm (right). **(E,F)** TUNEL staining was used for detecting apoptosis (red) of myocytes and DAPI (blue) was for nuclear staining. OA could significantly reduce the myocytes apoptosis. The scale bar represented 20 μm. Data are mean ± SD; *n* = 3–4/group. **(G)** Representative immunofluorescent images were presented to determine capillary density in ischemic muscles on day 28. Capillaries were stained with lectin (red) and nuclei were stained with DAPI (blue). The white arrowheads showed the region of blood vessels. The scale bar represented 50 μm. **(H)** Quantification of capillary density was expressed as lectin positive number per randomly chosen high-power field (HPF). *n* = 3/group. **(I)** Capillaries were stained with lectin (red), α-SMA (green), and nuclei were stained with DAPI (blue). The white arrowheads showed the region of mature, functional blood vessels. The scale bar represented 25 μm. **(J)** Quantification of the mature, functional blood vessels in the adductor muscle was expressed as lectin, α-SMA double-positive number per randomly chosen HPF. Data are mean ± SD; *n* = 5/group, ^#^ *p* < 0.05, ^##^ *p* < 0.01 *vs*. saline.

Mice undergoing HLI showed a time-dependent increase in tissue perfusion that reached about 30% restoration of blood flow by day 14 post-HLI. The mean ratios of ischemic to non-ischemic perfusion in the OA group (0.59 ± 0.14) and Sim-treated mice (0.52 ± 0.13) were significantly higher than that of saline group (0.33 ± 0.10). The recovery was more pronounced by day 28. OA treatment significantly accelerated perfusion recovery as compared with saline (0.71 ± 0.16 versus 0.45 ± 0.10), similar with the effect of Sim (0.63 ± 0.13), which showed that OA improved hindlimb ischemia in limb ischemic mice (Figures 1A,B). The H and E-stained sections of the gastrocnemius muscle shown in Figures 1C,D were used for the analysis of skeletal muscle remodeling, damage, and necrosis. Muscle regeneration was assessed by the number of myocytes with centrally located nuclei. The histopathological features of necrosis were defined by the presence of multi-cellular infiltrates and hyper-eosinophilic muscle cells that were devoid of nuclei. In the model mice treated with saline, the myofibers were shrunken and the intermuscular gaps were large. In the OA-treated groups, the muscles had repopulated with intensely staining myocytes and no obvious infiltration of inflammatory cells, indicating that OA reduced tissue injury and made skeletal myocytes regenerated. The TUNEL results showed that OA significantly reduced the gastrocnemius muscle cell apoptosis. This further demonstrated that OA could protect the muscle tissue surrounding the ischemic site (Figures 1E,F). In addition, the histological findings correlated with vascular and functional outcomes. The capillary density was assessed by quantification of lectin positive vessels observed in the interface between the skeletal muscles at day 28 post-ischemia. The level of capillary density was higher in the OA-treated mice compared with saline (Figures 1G,H). To determine the collateral formation and demonstrate the arterial nature of the newly formed vessels in the adductor muscle, we co-stained α-SMA and examined the number of tube-like structures, as shown in Figures 1I,J. Remarkably, the level of mature vessels was higher in the OA-treated mice compared with saline. Collectively, these results demonstrated that OA accelerated perfusion recovery, reduced tissue injury, and promoted angiogenesis after HLI.

### Oroxylin A Regulated the Secretion of Vascular Endothelial Growth Factor A, Angiopoietin-2, Fibroblast Growth Factor-2, and Platelet Derived Growth Factor BB at Distinct Time Points After Hind Limb Ischemia

**FIGURE 2:**
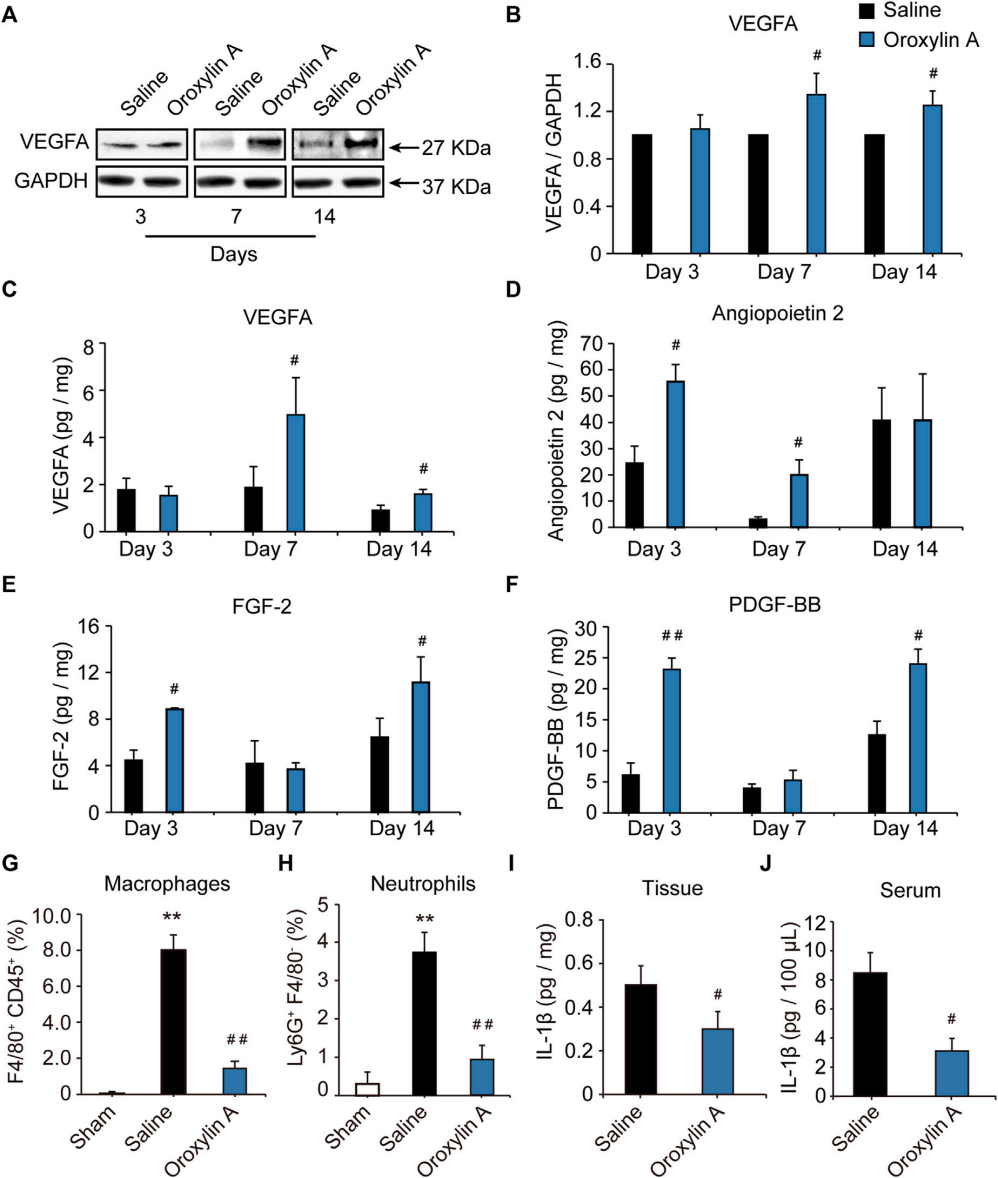
OA regulated the secretion of VEGFA, ANG-2, FGF-2, and PDGF-BB at distinct time points during angiogenesis after HLI and resolved inflammation during HLI. Lysates were immunoblotted with VEGF antibody, and detected with an enhanced chemiluminescence kit. Blots were normalized by densities for GAPDH as an internal control **(A,B)**. Quantitative VEGFA **(C)**, ANG-2 **(D)**, FGF-2 **(E)**, and PDGF-BB **(F)** in ischemic tissue at 3, 7 and 14 days after HLI in the presence of OA or saline were detected by ELISA. Values were represented as means ± SD (*n* = 3–4). Quantification by flow cytometry of F4/80^+^CD45^+^ macrophages **(G)** and Ly6G^+^F4/80^-^ neutrophils **(H)** in skeletal muscle isolated from mice undergoing HLI (day 3) and treated with saline or OA was presented. Tissue **(I)** and Serum **(J)** levels of IL-1β in mice undergoing HLI and treated with saline or OA were detected. Data were represented as mean ± SD (*n* = 5). **p < 0.01 *vs*. sham; ^#^ *p* < 0.05, ^##^ *p* < 0.01 *vs*. saline.

The formation of a mature vascular network requires the precise spatial and temporal regulation of many angiogenic factors, including VEGF, ANG-2, FGF-2, and PDGF. VEGF aids in vascular permeability and the recruitment of ECs, FGF-2 activates ECs proliferation and migration, while PDGF stimulates vascular stability (Carmeliet and Jain, 2011; Bai et al., 2018). To define the molecular mediators of angiogenesis secreted from ischemic tissue; the proteome antibody array was used to detect the regulated proteins in the gastrocnemius muscle post ischemia. OA modulated several secreted factors, including VEGFA, ANG-2, FGF-2, and PDGF-BB (Supplementary Figure S1A-C,S2). These regulated factors were correlated with the immune system and functions of cell migration, cell proliferation, and angiogenesis. (Supplementary Figure S1D-I). Further experiments were performed to identify the array results. Results showed that VEGFA levels were comparable between groups at the 3-days time point, while OA improved VEGFA levels at day 7 and 14 after HLI, when compared with the respective saline group (Figures 2A–C). This facilitated vessel sprouting and lumen elongation during angiogenesis (Potente et al., 2011). In addition, OA significantly increased ischemia-induced decreases in ANG-2 at days 3 and 7 after HLI (Figure 2D). These results were consistent with the defined role of OA in regulating pericytes that detach from the vessel wall to enlarge the size of the lumen (Carmeliet and Jain, 2011). ANG-2 levels were comparable between groups at the day 14 time point. FGF is critical for maintaining vascular integrity, because inhibition of FGFR signaling in ECs leads to vascular disintegration (Murakami et al., 2008). After HLI, FGF was increased in the OA group at days 3 and 14, as compared with saline group. However, there was no significant difference between the two groups 7 days after HLI (Figure 2E). Damaged PDGF-BB might lead to vascular leakage, tortuosity, microaneurysm formation, and bleeding due to lack of pericytes, which is not conducive to the repair of ischemic injury. PDGF-BB plays a key role in the mature stage of angiogenesis (Skovseth et al., 2005; Mittermayr et al., 2016). Like FGF, PDGF-BB was also increased in the early and late stages of angiogenesis when compared with the respective saline group and PDGF-BB levels were comparable between groups at 7 days (Figure 2F). Current research revealed that angiogenic factors go through a dynamic progression in this multi-step angiogenesis process, in which tubular structures are created, elongated, and then matured during angiogenesis after HLI. In addition, OA promoted angiogenesis by regulating VEGFA, ANG-2, FGF-2, and PDGF-BB at distinct time points after HLI.

### Oroxylin A Resolved Inflammation During Hind Limb Ischemia

The inflammatory response is an integral part of the ischemic microenvironment, which is tightly coupled to angiogenesis. OA has been reported to have strong anti-inflammatory effects in macrophages *in vitro*. Next, we sought to determine how OA modulated the inflammatory microenvironment *in vivo*. In comparison with sham group, levels of CD45^+^F4/80^+^ macrophages (Figure 2G) and Ly6G^+^F4/80^-^ neutrophils (Figure 2H) increased significantly in skeletal muscle of mice undergoing HLI. This increase was prevented in mice treated with OA. Meanwhile, OA significantly decreased the ischemia-induced increase in pro-inflammatory cytokine IL-1β (Figures 2I,J). These results indicated that OA resolved inflammation by reducing macrophage and neutrophil recruitment and proinflammatory cytokine production, which will have a positive effect on promoting blood flow recovery.

### Oroxylin A Promoted Release of Vascular Endothelial Growth Factor A and Angiopoietin-2 in Skeletal Muscle Cells

**FIGURE 3:**
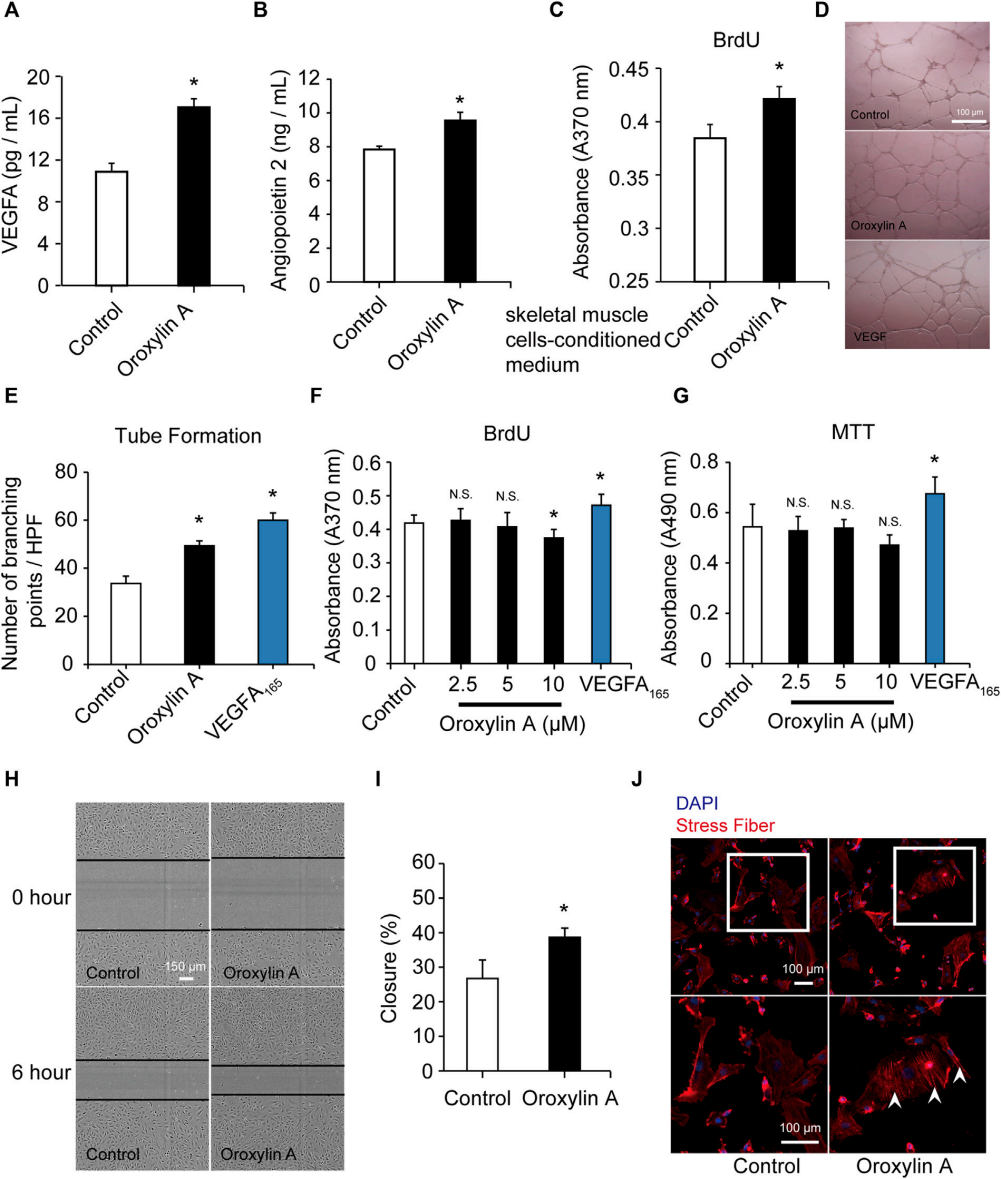
OA promoted the secretion of VEGFA and ANG-2 in skeletal muscle cells and induced tube formation and migration in HUVECs. Skeletal muscle cells were cultured with DMSO or OA (2.5 µM) for 24 h, and then the contents of VEGFA **(A)** and ANG-2 **(B)** in the supernatant were quantified by ELISA. After DMSO or OA treatment, medium derived from skeletal muscle cells was added to HUVEC in culture for 24 h, and then assayed by a BrdU kit **(C)**. Data were represented as mean ± SD (*n* = 6). Tube formation of HUVECs were performed on Matrigel in response to DMSO, OA (2.5 μM) or VEGFA_165_ for 18 h, with VEGFA_165_ as a positive control. The scale bars represented 100 μm **(D)**. Quantification of tube formation were presented as several branching points **(E)**. HUVECs were cultured with DMSO or OA (2.5, 5, 10 µM) for 24 h, and then assayed by a BrdU kit **(F)** and MTT assay **(G)**. VEGFA_165_ was acted as a positive control. Representative images and quantification of ECs migration following scrape injury in response to DMSO or OA (2.5 μM) were shown in **H and I**. The scale bars represented 150 μm. Actin cytoskeleton rearrangement in HUVECs was shown in **(J)**. Below is a higher magnification of the white-boxed area. Formation of stress fibers terminated at pointed edges (white arrowhead). The scale bars represented 100 μm. Data were represented as mean ± SD (*n* = 5). *p < 0.05, N.S. not significant, *vs.* control.

Skeletal muscle is a secretory organ that can secrete various angiogenic factors, and is very crucial for angiogenesis. We examined the effect of the OA-treated secretome of skeletal muscle cells on vascular endothelial cells. OA (2.5 μM) significantly promoted the release of VEGFA (Figure 3A) and ANG-2 (Figure 3B) in skeletal muscle cells. After OA treatment, the conditioned medium derived from skeletal muscle cells was added to HUVEC in culture for 24 h. There was a statistically significant increase in the pro-proliferative activity of ECs as compared to conditioned medium after DMSO intervention (Figure 3C). This study demonstrated that OA-mediated angiogenesis was associated with increase of angiogenic factors within the skeletal muscle microenvironment.

### Oroxylin A Induced Tube Formation and Migration in Human Umbilical Vein Endothelial Cells

*In vitro* angiogenesis assays were conducted. EC proliferation and migration contributes to dissemination of pre-existing vessels to form new vessels. OA also significantly induced the formation of capillary-like structures (Figures 3D,E). OA did not stimulate EC proliferation, which was robustly stimulated by VEGFA_165_ (100 ng/ml). This lack of effect on proliferation was not due to a decrease in cell viability (Figures 3F,G; Supplementary Figure S3). We further investigated the effect of OA on cell migration and it was observed that OA directly enhanced EC migration following a scrape injury (Figures 3H,I). OA-induced rearrangement of the actin cytoskeleton and enhanced the formation of stress fibers. These stress fibers terminated at pointed edges and were a typical morphological feature in migrating cells (Figure 3J). These results indicated that OA stimulated the migration of HUVECs.

### Oroxylin A Induced Endothelial Cell Migration Through the Ras Homolog Gene Family Member A/Rho-Associated Coiled-Coil Kinase II Pathway

**FIGURE 4:**
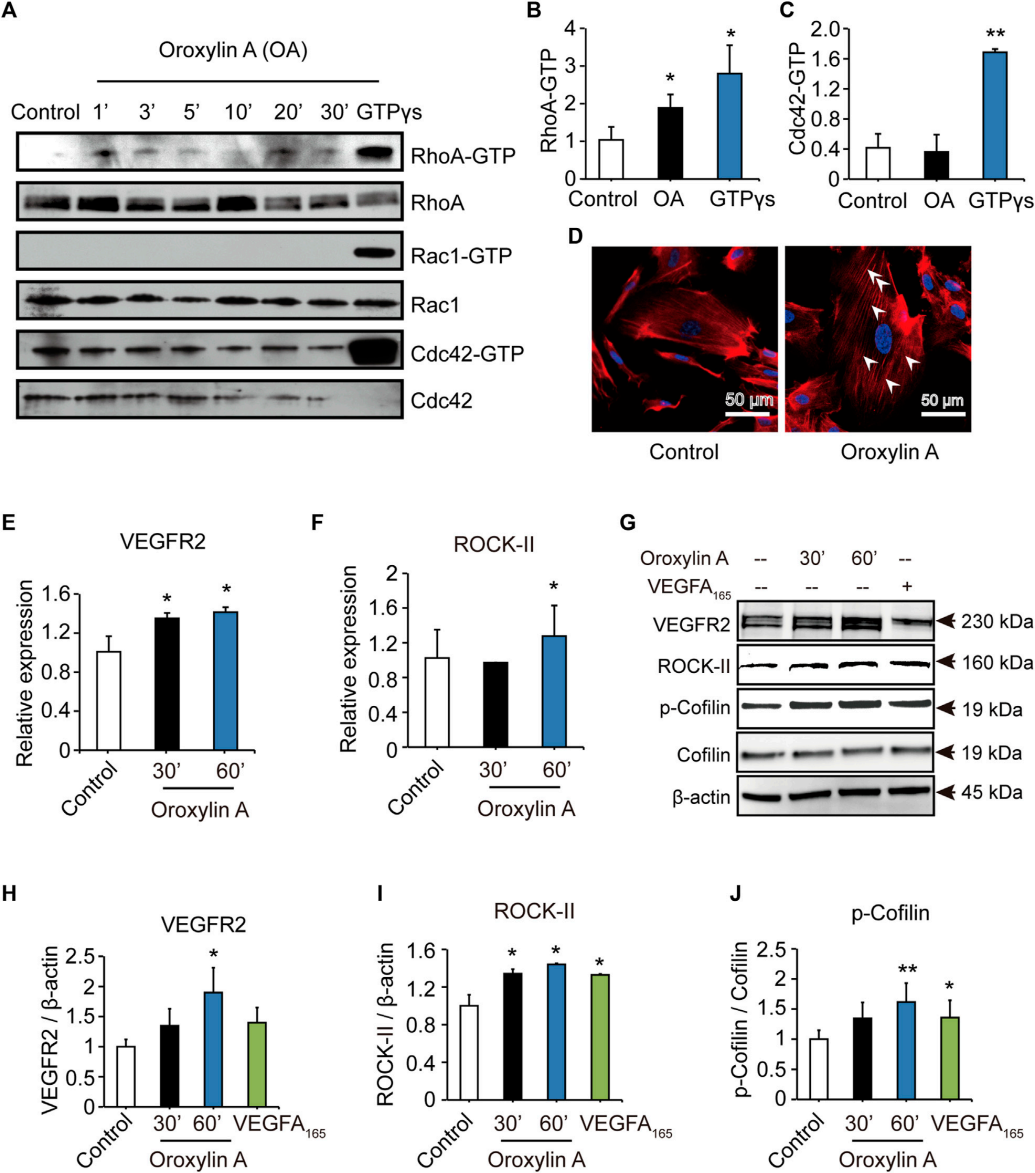
OA induced stress fiber formation and cell migration in HUVECs through RhoA/ROCK-II pathway. The amounts of active GTP-bound RhoA (RhoA-GTP, rhotekin-RBD), Rac1 (Rac1-GTP, PAK-PBD), Cdc42 (Cdc42-GTP, PAK-PBD) were determined by a pull-down assay, in non-stimulated (control) or OA-stimulated (2.5 μM) HUVECs for 1 min (1′), 3′, 5′, 10′, 20′ or 30’. Total RhoA, Rac1, Cdc42 in total cell extracts were also detected **(A)**. Quantification of active, GTP-bound state was presented as RhoA-GTP (20′) **(B)** and Cdc42-GTP (3′) **(C)**. HUVECs treated for 20 min with 2.5 μM OA after serum were starved and subsequently stained with rhodamine-phalloidin and with DAPI for nuclei. RhoA induced actin stress fibers were visible **(D)**. *VEGFR2* **(E)** and *ROCK-II* **(F)** mRNA expression in HUVECs were stimulated with 2.5 μM OA for 30, 60 min. HUVECs stimulated with DMSO, 2.5 μM OA for 30, 60 min and VEGFA_165_ for 60 min. Western blotting analysis of VEGFR2, ROCK-II or *p*-Cofilin and total Cofilin in HUVECs were performed. β-actin expression was presented as an internal control of protein loading. VEGFA_165_ was acted as a positive control **(G)**. Quantitative data included VEGFR2 **(H)**, ROCK-II **(I)** and *p*-Cofilin **(J)**. Data were represented as mean ± SD (*n* = 3). *p < 0.05; **p < 0.01 *vs.* control.

To identify the mechanism of OA, we examined Rho GTPases, which play a fundamental role in EC migration (Warner et al., 2019). OA increased the amount of active, GTP-bound RhoA, but did not increase the amount of Cdc42 or Rac1. In addition, OA induced the formation of stress fiber mediated by RhoA (Figures 4A–D). Previous studies have demonstrated that VEGFR2 activation induced the stimulation of RhoA and its major downstream effector, Rho-associated protein kinase ROCK-II, to regulate stress fiber formation and cell migration in ECs (Bryan et al., 2010; Liu et al., 2018). In addition, Cofilin has been previously shown to regulate cell migration (Li et al., 2015). Our data showed that OA increased the expression of VEGFR2, ROCK-II, and increased the phosphorylation of Cofilin in HUVECs in a time-dependent manner (Figures 4E–J).

**FIGURE 5:**
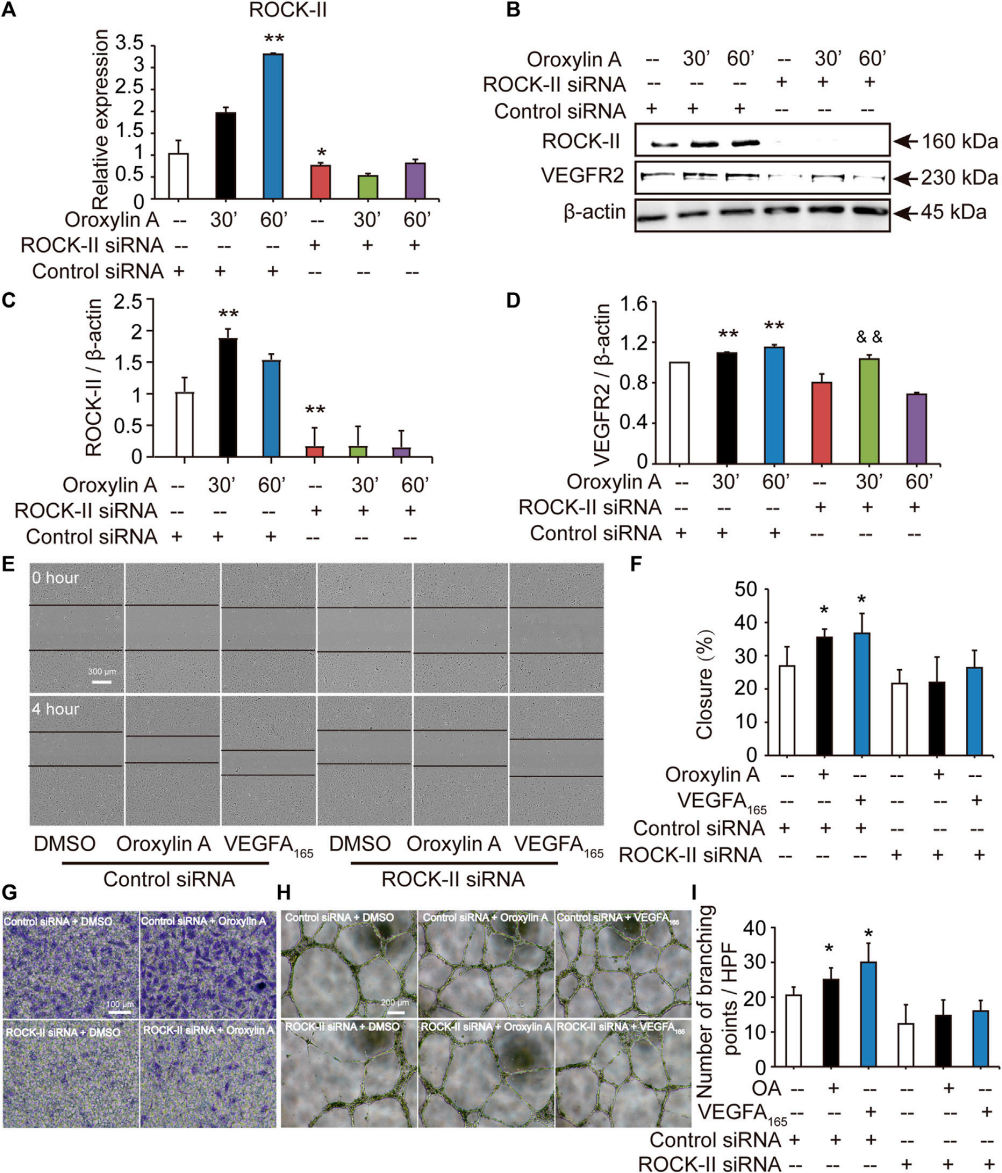
Cell migrations and capillary-like structures of HUVECs induced by OA were decreased by silencing ROCK-II with siRNA. HUVECs were transfected with non-specific siRNA or ROCK-II siRNA and stimulated with DMSO or 2.5 μM OA for 30, 60 min and analyzed using qRT-PCR **(A)**. Western blotting analysis of ROCK-II and VEGFR2 in HUVECs transfected with non-specific siRNA or ROCK-II siRNA and stimulated with DMSO or 2.5 μM OA for 30, 60 min were performed in **(B)**. Quantitative data included ROCK-II **(C)** and VEGFR2 **(D)**. The mobility of HUVECs was analyzed by using scrape injury, with VEGFA_165_ as a positive control. HUVECs were transfected with control siRNA or ROCK-II siRNA and stimulated with DMSO, 2.5 μM OA or VEGFA_165_ for 4 h **(E)**. Quantitative data were shown in **(F)**. Performed Transwell invasion of HUVECs transfected with control siRNA or ROCK-II siRNA and stimulated with DMSO or 2.5 μM OA were presented in **(G)**. Representative images of tube formation of HUVECs transfected with control siRNA or ROCK-II siRNA and stimulated with DMSO, 2.5 μM OA or VEGFA_165_ were presented, with VEGFA_165_ as a positive control **(H)**. Quantitative data were shown in **(I)**. Data were represented as mean ± SD (*n* = 3). *p < 0.05; **p < 0.01 *vs*. control siRNA, ^&&^ *p* < 0.01 *vs*. ROCK-II siRNA.

ROCK-II is the downstream of VEGFR2. It is estimated that knockdown of ROCK-II will not alter the expression of VEGFR2. We tested the level of VEGFR2 after siROCK-II. Figures 5A–D showed that the transfection of ROCK-II siRNA resulted in a marked reduction in ROCK-II. After siROCK-II treatment, the level of VEGFR2 was significantly increased for 30 min after OA treatment, while after 60 min of OA treatment, the level of VEGFR2 was not altered, which is not in line with the expected results. Furthermore, the results showed that the concurrent cell migrations and capillary-like structures of HUVECs that were induced by OA treatments were able to be decreased by silencing ROCK-II (Figures 5E–I). This indicated that ROCK-II played an important role in OA-induced stress fiber formation and EC migration.

### Oroxylin A Regulated the T-Box20/Prokineticin 2 Signaling Pathway

**FIGURE 6:**
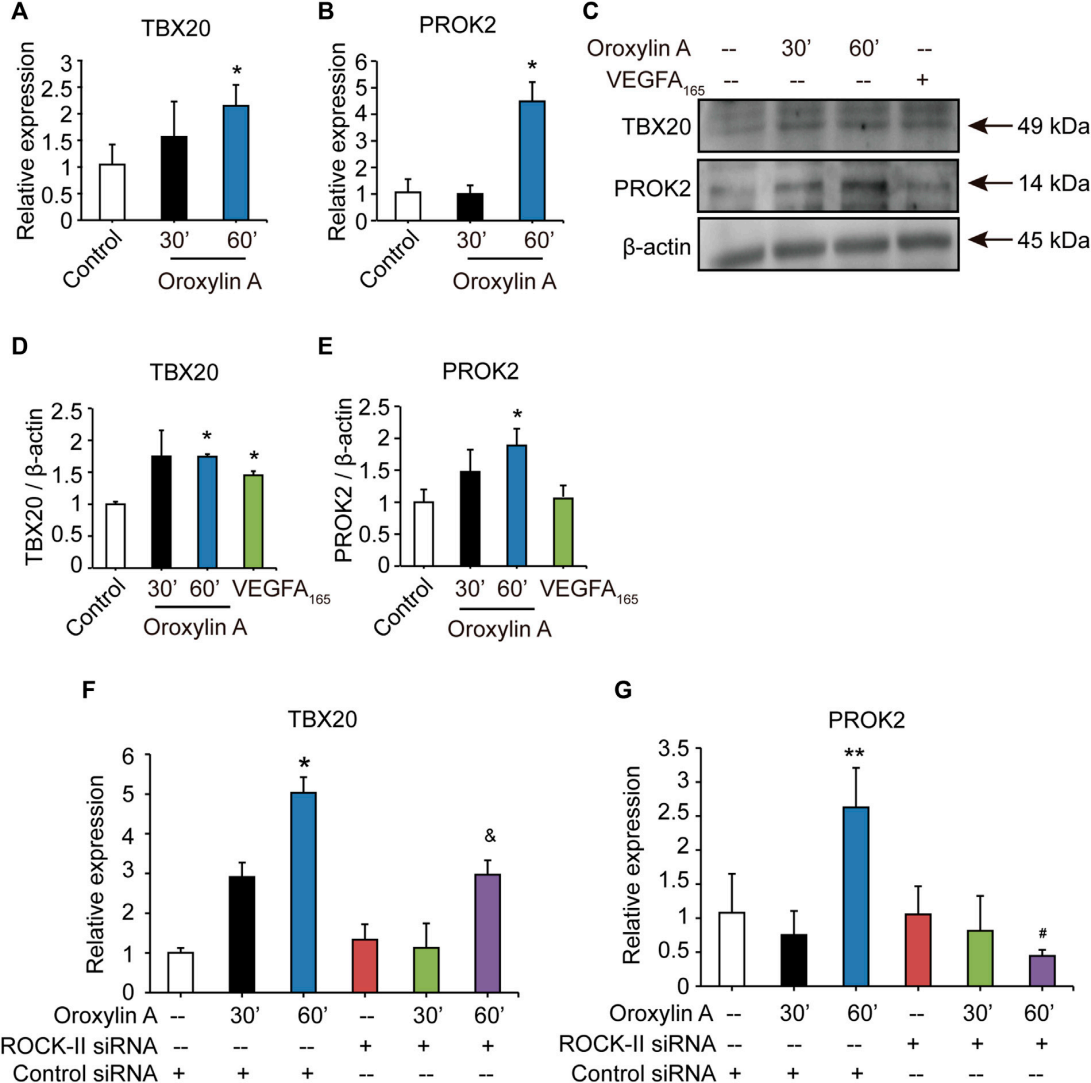
OA regulated TBX20/PROK2 signaling pathway. Western blotting analysis of TBX20 or PROK2 in HUVECs stimulated with DMSO, 2.5 μM OA (30, 60 min) and VEGFA_165_ (100 ng/ml), β-actin expression an internal control of protein loading was performed, with VEGFA_165_ as a positive control **(A)**. Quantitative data included TBX20 **(B)** and PROK2 **(C)**. *TBX20* **(D)** and *PROK2* **(E)** mRNA expression in HUVECs treated with DMSO or 2.5 μM OA for 30, 60 min were analyzed using qRT-PCR. Statistical analysis of *TBX20* **(F)** and *PROK2* **(G)** mRNA of HUVECs transfected with non-specific siRNA or ROCK-II siRNA and stimulated with DMSO or 2.5 μM OA for 30, 60 min were presented. Data were represented as mean ± SD (*n* = 3). *p < 0.05, **p < 0.01 *vs*. control siRNA; ^&^ *p* < 0.05 *vs*. ROCK-II siRNA; ^#^ *p* < 0.05 *vs*. control siRNA + OA 60 min.

After silencing ROCK-II with siRNA, we observed that the concurrent cell migrations and capillary-like structures of HUVECs were significantly decreased, but still existed. Therefore, we speculated that OA may regulate other key pathways to promote angiogenesis. Previous studies have shown that loss of TBX20-PROK2 hindered tube formation and EC migration in HUVECs (Meng et al., 2018). To verify the hypothesis, we explored the role of OA on the TBX20/PROK2 signaling pathway. Both qRT-PCR and Western blot analysis showed that OA significantly increased TBX20 and PROK2 expression in HUVECs (Figures 6A–E). These results indicated that OA regulated the TBX20/PROK2 signaling pathway. Transfection of ROCK-II siRNA did not affect TBX20 and PROK2 expression when compared with the control siRNA, while PROK2 was robustly decreased by OA after silencing ROCK-II with siRNA (Figures 6F,G). These results confirmed that PROK2 functions downstream of ROCK-II. The above results suggest that these two pathways may play essential roles in promoting EC migration and that OA could promote EC migration through these two pathways to promote angiogenesis. Further investigation is required to fully understand this detailed mechanism.

## Discussion

In this study, we investigated the therapeutic efficacy of OA for the treatment of PAD. OA treatment significantly accelerated perfusion recovery following 14 days of therapy. In addition, OA treatment enhanced angiogenesis by inducing EC migration, promoted the secretion of VEGFA and ANG-2 in skeletal muscle cells, and modulated the inflammatory microenvironment. These results suggest that OA is emerging as a novel approach for patients with PAD.

In recent research, pivotal regulators of angiogenesis have been tested in human clinical trials to promote angiogenesis; however therapeutic outcomes remain far from satisfactory. Clinical designs for these trials have been single-targeted and have not considered the entire mechanism of angiogenesis, like the sprouting of ECs and further vascular maturation (Roncalli et al., 2008; Minoshima et al., 2018). VEGFA promotes tip cell selection, stalk elongation, and vascular maintenance (Carmeliet and Jain, 2011). FGF is critical for maintaining vascular integrity (Carmeliet and Jain, 2011). PDGF-BB plays a key role in the mature stage of angiogenesis (Carmeliet and Jain, 2011). Sequential delivery of VEGF and PDGF-BB improves revascularization and heart function after myocardial infarction (Awada et al., 2015). FGF-2 and PDGF-BB alone may cause vascular degeneration, while the combination of the two is more conducive to vascular stability and maturation (de Paula et al., 2009; Li et al., 2010). Sequential delivery of VEGF, FGF-2, and PDGF resulted in significant angiogenic differentiation of HUVECs and rapid formation of mature vascular networks in the chorioallantoic membrane (Bai et al., 2018). Nintedanib, co-targeting PDGFRs, VEGFRs, and FGFR, have been used in phase 3 trials (Scagliotti et al., 2019). In this study, OA promoted angiogenesis by regulating VEGFA, ANG-2, FGF-2, and PDGF-BB at distinct time points during angiogenesis. This implies that OA therapy could be exploited as a promising strategy to promote angiogenesis in clinic.

In this study, murine HLI model was performed on C57BL/6 mice as previously described (Limbourg et al., 2009; Brenes et al., 2012; Zhang et al., 2016; Ministro et al., 2017; Aref et al., 2019; Chang et al., 2019). Although there is minimal variation of the gross vascular anatomy of the femoral artery and its main branches in all mouse strains, the murine genetic background greatly influences the vascular response and the recovery of perfusion after femoral artery ligation. C57BL/6 mice are known to recover quickly from ischemia, as compared with the slow recovering Balb/C mice. It is critical to choose the optimal timing for perfusion imaging and tissue harvest. In our research, mice undergoing HLI showed a time-dependent increase in perfusion ratio that reached about 30% restoration of blood flow by day 14 post-HLI. The mean ratios of ischemic to non-ischemic perfusion in the OA group (0.59 ± 0.14) were significantly higher than that of saline group (0.33 ± 0.10), indicating OA improved perfusion in HLI mice. To further confirm that OA is an effective compound for treating HLI, animal experiments using other mouse strains will be performed in the future studies.

In the traditional Chinese medicinal practice, most crude drugs or compound formulas are prepared as decoctions and taken orally. Pharmacokinetic studies of OA after intragastrical administration in rats indicated that OA can be absorbed from the gastrointestinal tract in its native form and the concentration of OA in the plasma increased with time (Li et al., 2011; Cai et al., 2016; Ren et al., 2020). OA has two metabolites: oroxylin A 7-O-glucuronide (OG) and oroxylin A sodium sulfonate (OS). After the oral administration of OA, this molecule was more widely distributed in tissue than its metabolites and the tissue concentration level of OA was the highest. The C_max_ of OA was lower and the elimination rate was slower than intravenous administration. The angiogenic effects of active metabolites of OA will be further investigated in future studies.

Several therapeutic strategies have been tested to increase angiogenesis, including the use of growth factors and mononuclear cells. These approaches have had limited success in large clinical trials, which could be related to the chronic inflammatory environment typically encountered in patients with PAD (Cooke and Losordo, 2015; Zhang et al., 2016). Biopsies from patients with PAD showed clear signs of inflammation, with a significantly higher density of macrophages (Caradu et al., 2018). The inflammatory response is an integral part of the ischemic microenvironment, which is tightly coupled to angiogenesis (Jalkanen et al., 2016). Inflammatory cells multitask at the ischemic site by facilitating cellular debris removal and producing chemokines, metabolites, and growth factors. These molecules are helpful for the repair of ischemic tissue damage. Unfortunately, this well-orchestrated response becomes dysregulated in PAD. The ischemic tissue can produce a continuous inflammatory response, resulting in impaired tissue function. Previous studies have shown that OA had strong anti-inflammatory effects on macrophages *in vitro* (Wang et al., 2013). Our data revealed that, levels of neutrophils and macrophages increased significantly in the skeletal muscle of mice undergoing HLI. In addition, OA resolved inflammation by reducing neutrophil and macrophage recruitment and down-regulating pro-inflammatory cytokine IL-1β production in serum and tissue. OA could also promote ischemic tissue repair by promoting angiogenesis and downregulating the number of inflammatory cells and the secretion of inflammatory factors. These findings offered a novel beneficial mechanism of OA regarding PAD protection.

Angiogenesis requires EC proliferation and migration. Unlike growth factors, OA selectively controlled EC migration, but did not stimulate EC proliferation. Since we utilized VEGF as positive control, cell proliferation was significantly enhanced in VEGF group, indicating the experimental system is credible. There are several possible explanations for this unexpected result. First, the OA-mediated elevated expression of VEGF in HUVEC was not sufficient to promote proliferation of ECs. Second, OA may inhibit proliferation of HUVEC through undiscovered mechanisms. This inhibitory effect counteracted the mitogenic effects of OA mediated by VEGF upregulation. Third, the concentrations, duration of drug delivery, and observation time may be responsible for the property of OA revealed in the present study. Further investigation is needed to unravel the detailed mechanisms of OA’s effects.

Other preclinical studies have showed that inhibition of RhoA could prevent VEGF-enhanced EC migration in response to vascular injury (Van Nieuw Amerongen et al., 2003). Our study demonstrated that OA induced stress fiber formation and EC migration associated with an increase in RhoA activation, implying that RhoA may act as a possible therapeutic target of OA for inducing EC migration. ROCK-II is a downstream effector of RhoA. In response to VEGF, ECs with ROCK-II knockdown exhibit a drastic reduction in migration and tube formation (Liu et al., 2018). OA was found to stimulate ROCK-II expression. In HUVECs lacking ROCK-II, the beneficial effects of OA were abrogated. These data indicated that OA promoted tube formation through a ROCK-II-dependent mechanism. ROCK-activated LIMK then phosphorylates Cofilin and inactivates its actin-depolymerization activity, leading to stabilization of actin filaments (Li et al., 2005; Li et al., 2015). OA can upregulate the phosphorylation at serine-3 of Cofilin, which suggests that OA might promote stabilization of actin filaments by phosphorylating Cofilin. Therefore, it is reasonable to assume that the enhanced RhoA/ROCK-II/p-Cofilin signaling pathway induced by OA is associated with stress fiber formation and therefore improves tube formation in HUVECs.

During EC migration, in addition to the RhoA/ROCK-II signaling pathway, the TBX20/PROK2 signaling pathway acts as a “biological capacitor” to relay and sustain the pro-angiogenic effect of VEGF. This may be an aspect worth exploring for the treatment of PAD (Meng et al., 2018). TBX20 encodes a key transcription factor that is expressed in the heart, eyes, ventral neural tube, and limbs during embryonic development (Meins et al., 2000). PROK2 is a secreted protein that functions downstream of TBX20 and helps to regulate angiogenesis. A recent study showed that TBX20-regulated tube formation and cell migration in ECs, along with intramuscular injection of recombinant PROK2 protein increased blood perfusion recovery in the ischemic limb (Meng et al., 2018). Our present study validated the upregulation of TBX20 and PROK2 after treatment with OA in HUVECs, indicating that OA may promote EC migration and tube formation by promoting the TBX20/PROK2 signaling pathway. Previous work demonstrated that the regulation of GTPase activity is implicated in the modulation E-cadherin expression through a pathway involving TBX (Yano et al., 2011). ROCK inhibition resulted in a drastic change in colony morphology, accompanied by the loss of TBX20 in murine embryonic stem cells and the induction of pluripotent stem cells (Cheng et al., 2015). We evaluated whether ROCK-II regulates the TBX20/PROK2 signaling pathway after OA treatment. The results showed that transfection of ROCK-II siRNA did not affect TBX20 and PROK2 expression; however, PROK2 was robustly decreased by OA after silencing ROCK-II. For the first time, we observed that PROK2 may be regulated by ROCK-II during angiogenesis *in vitro* after OA treatment. Further studies will be performed to determine whether ROCK-II affects PROK2 expression in the OA-treated group during angiogenesis after HLI. These results provide new drug targets to treat diseases with dysregulated angiogenesis.

Previous studies have shown that pathologic neovascularization is inhibited by OA (Gao et al., 2010; Song et al., 2012; Zhang et al., 2018; Zhao et al., 2018). The effect of OA differs between different studies due to different concentrations and the complexity of various tissue microenvironment. Previous studies have shown that HUVECs cultured in medium 199 containing FBS, EGF and endothelial cell growth supplement, which were treated with OA for 1 h at concentrations of 1, 10, and 100 μM, were able to suppress the VEGF and LPS-stimulated migration and tube formation. And OA (1 μM) suppressed the VEGF-stimulated migration and tube formation through blocking MAPK signaling pathway induced by VEGF. In murine primary liver sinusoidal endothelial cells (LSECs), concentrations of 20, 30, and 40 μM of OA inhibited hypoxia-induced angiogenesis as indicated by MTT and tube formation assays. For 24 h of treatment with oroxylin A-7-glucuronide (Oroxyloside), a main metabolite of OA, inhibited the proliferation, migration, and tube formation of human endothelial cells at concentrations of 40, 80, and 160 μM. Compared with previous studies, we explored the direct effect of OA on endothelial cells. The concentrations we chose are 2.5, 5, 10 and 20 μM. The treatment time is 24 h. The results showed that OA inhibited the viability of endothelial cells at 20 μM and inhibited endothelial cell proliferation at 10 μM after 24 h of treatment. A concentration of 2.5 μM OA was found to significantly induce tube formation in HUVECs. We selected 2.5 μM as the concentration of OA in subsequent experiments. OA promoted endothelial cell migration through the GTPases signaling pathway. The above results indicated that OA may play a dual role: low concentration of OA promotes angiogenesis, while high concentration of OA possesses anti-angiogenic effects. This is not uncommon, and a similar phenomenon took place in tetramethyl pyrazine (TMP), *panax ginseng*, rhubarb, and danshensu (Zhang et al., 2010; Qin et al., 2011; Chen et al., 2012; Zhang et al., 2014; Yin et al., 2017), implying that drug dose is an important parameter for the efficacy of drugs and that the same drug may play a diametrically opposite and context-dependent role in different pathological states. This property of OA could be particularly beneficial in the context of atherosclerosis and cancer, where angiogenesis could fuel disease progression.

**FIGURE 7:**
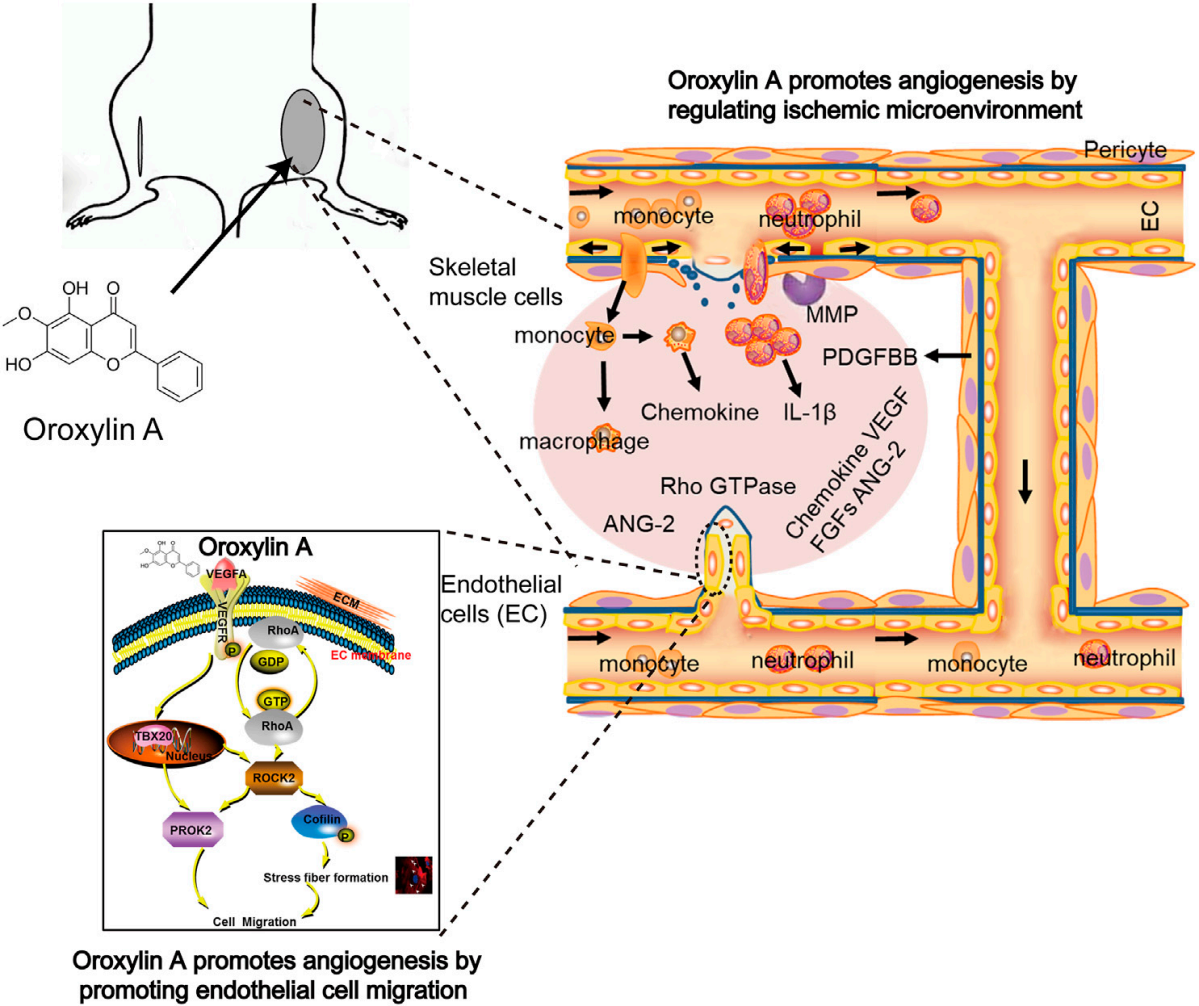
OA increased angiogenesis through multiple mechanisms involving enhanced EC migration, elevated angiogenic factors release, and induced stabilization and growth of blood vessels.

In conclusion, the data suggested that OA increased angiogenesis through multiple mechanisms (Figure 7). First, OA could act on ECs to stimulate endothelial migration. Second, OA treatment increased the expression of several angiogenic factors, including VEGFA, ANG-2, PDGF-BB, and FGF-2 in ischemic muscle at distinct time points post ischemia. Third, OA promoted the release of VEGFA and ANG-2 in skeletal muscle cells, which induced proliferation of ECs. Finally, OA promoted the formation of smooth, muscle-covered, mature blood vessels. The maturation of blood vessels into multilayer structures is essential for their persistence. These results uncovered a new role of OA in tissue revascularization following ischemic injury and could open up new directions for the development of novel therapeutic interventions for patients with PAD.

## Data Availability

The original contributions presented in the study are included in the article/Supplementary Material, further inquiries can be directed to the corresponding author.

## References

[B1] Albrecht-Schgoer K. Barthelmes J. Schgoer W. Theurl M. Nardin I. Lener D. (2017). Nanoparticular Delivery System for a Secretoneurin Derivative Induces Angiogenesis in a Hind Limb Ischemia Model. J. Controlled Release 250, 1–8. 10.1016/j.jconrel.2017.02.004 28167285

[B2] Aref Z. de Vries M. R. Quax P. H. A. (2019). Variations in Surgical Procedures for Inducing Hind Limb Ischemia in Mice and the Impact of These Variations on Neovascularization Assessment. Ijms 20 (15), 3704. 10.3390/ijms20153704 PMC6696155 31362356

[B3] Awada H. K. Johnson N. R. Wang Y. (2015). Sequential Delivery of Angiogenic Growth Factors Improves Revascularization and Heart Function after Myocardial Infarction. J. Controlled Release 207, 7–17. 10.1016/j.jconrel.2015.03.034 PMC4430430 25836592

[B4] Bai Y. Bai L. Zhou J. Chen H. Zhang L. (2018). Sequential Delivery of VEGF, FGF-2 and PDGF from the Polymeric System Enhance HUVECs Angiogenesis *In Vitro* and CAM Angiogenesis. Cell Immunol. 323, 19–32. 10.1016/j.cellimm.2017.10.008 29111157

[B5] Brenes R. A. Jadlowiec C. C. Bear M. Hashim P. Protack C. D. Li X. (2012). Toward a Mouse Model of Hind Limb Ischemia to Test Therapeutic Angiogenesis. J. Vasc. Surg. 56 (6), 1669–1679. 10.1016/j.jvs.2012.04.067 22836102 PMC3508332

[B6] Bryan B. A. Dennstedt E. Mitchell D. C. Walshe T. E. Noma K. Loureiro R. (2010). RhoA/ROCK Signaling Is Essential for Multiple Aspects of VEGF‐mediated Angiogenesis. FASEB j. 24, 3186–3195. 10.1096/fj.09-145102 20400538 PMC2923346

[B7] Cai Y. Li S. Li T. Zhou R. Wai A. T.-S. Yan R. (2016). Oral Pharmacokinetics of Baicalin, Wogonoside, Oroxylin A 7- O -β- D -glucuronide and Their Aglycones from an Aqueous Extract of Scutellariae Radix in the Rat. J. Chromatogr. B 1026, 124–133. 10.1016/j.jchromb.2015.11.049 26809374

[B8] Caradu C. Couffinhal T. Chapouly C. Guimbal S. Hollier P.-L. Ducasse E. (2018). Restoring Endothelial Function by Targeting Desert Hedgehog Downstream of Klf2 Improves Critical Limb Ischemia in Adults. Circ. Res. 123, 1053–1065. 10.1161/circresaha.118.313177 30355159

[B9] Carmeliet P. Jain R. K. (2011). Molecular Mechanisms and Clinical Applications of Angiogenesis. Nature 473, 298–307. 10.1038/nature10144 21593862 PMC4049445

[B10] Chang T.-T. Lin L.-Y. Chen J.-W. (2019). Inhibition of Macrophage Inflammatory Protein-1β Improves Endothelial Progenitor Cell Function and Ischemia-Induced Angiogenesis in Diabetes. Angiogenesis 22 (1), 53–65. 10.1007/s10456-018-9636-3 29987448

[B11] Chen I.-J. Chang M.-Y. Chiao S.-L. Chen J.-L. Yu C.-C. Yang S.-H. (2012). Korean Red Ginseng Improves Blood Pressure Stability in Patients with Intradialytic Hypotension. Evidence-Based Complement. Altern. Med. 2012, 1–9. 10.1155/2012/595271 PMC3356894 22645630

[B12] Cheng Y.-T. Yeih D.-F. Liang S.-M. Chien C.-Y. Yu Y.-L. Ko B.-S. (2015). Rho-associated Kinase Inhibitors Promote the Cardiac Differentiation of Embryonic and Induced Pluripotent Stem Cells. Int. J. Cardiol. 201, 441–448. 10.1016/j.ijcard.2015.08.118 26313863

[B13] Cooke J. P. Losordo D. W. (2015). Modulating the Vascular Response to Limb Ischemia. Circ. Res. 116 (9), 1561–1578. 10.1161/CIRCRESAHA.115.303565 25908729 PMC4869986

[B14] de Paula E. V. Flores-Nascimento M. C. Arruda V. R. Garcia R. A. Ramos C. D. Guillaumon A. T. (2009). Dual Gene Transfer of Fibroblast Growth Factor-2 and Platelet Derived Growth Factor-BB Using Plasmid Deoxyribonucleic Acid Promotes Effective Angiogenesis and Arteriogenesis in a Rodent Model of Hindlimb Ischemia. Translational Res. 153, 232–239. 10.1016/j.trsl.2009.02.002 19375684

[B15] Fan Y. Lu H. Liang W. Garcia-Barrio M. T. Guo Y. Zhang J. (2018). Endothelial TFEB (Transcription Factor EB) Positively Regulates Postischemic Angiogenesis. Circ. Res. 122 (7), 945–957. 10.1161/CIRCRESAHA.118.312672 29467198 PMC5918429

[B16] Gao Y. Lu N. Ling Y. Chen Y. Wang L. Zhao Q. (2010). Oroxylin A Inhibits Angiogenesis through Blocking Vascular Endothelial Growth Factor-Induced KDR/Flk-1 Phosphorylation. J. Cancer Res. Clin. Oncol. 136 (5), 667–675. 10.1007/s00432-009-0705-2 19888602 PMC11828271

[B17] Gerhard-Herman M. D. Gornik H. L. Barrett C. Barshes N. R. Corriere M. A. Drachman D. E. (2017). 2016 AHA/ACC Guideline on the Management of Patients with Lower Extremity Peripheral Artery Disease: Executive Summary: A Report of the American College of Cardiology/American Heart Association Task Force on Clinical Practice Guidelines. Circulation 135, e686–e725. 10.1161/cir.0000000000000470 27840332 PMC5479414

[B18] Gorenoi V. Brehm M. U. Koch A. Hagen A. (2017). Growth Factors for Angiogenesis in Peripheral Arterial Disease. Cochrane Database Syst. Rev. 6, CD011741. 10.1002/14651858.CD011741.pub2 28594443 PMC6481523

[B19] Jalkanen J. Maksimow M. Hollmén M. Jalkanen S. Hakovirta H. (2016). Compared to Intermittant Claudication Critical Limb Ischemia Is Associated with Elevated Levels of Cytokines. PLoS One 11 (9), e0162353. 10.1371/journal.pone.0162353 27611073 PMC5017674

[B20] Lee H. Kang K.-T. (2018). Advanced Tube Formation Assay Using Human Endothelial Colony Forming Cells for *In Vitro* Evaluation of Angiogenesis. Korean J. Physiol. Pharmacol. 22 (6), 705–712. 10.4196/kjpp.2018.22.6.705 30402031 PMC6205943

[B21] Li C. Zhang L. Lin G. Zuo Z. (2011). Identification and Quantification of Baicalein, Wogonin, Oroxylin A and Their Major Glucuronide Conjugated Metabolites in Rat Plasma after Oral Administration of Radix Scutellariae Product. J. Pharm. Biomed. Anal. 54 (4), 750–758. 10.1016/j.jpba.2010.10.005 21051171

[B22] Li J. Wei Y. Liu K. Yuan C. Tang Y. Quan Q. (2010). Synergistic Effects of FGF-2 and PDGF-BB on Angiogenesis and Muscle Regeneration in Rabbit Hindlimb Ischemia Model. Microvasc. Res. 80, 10–17. 10.1016/j.mvr.2009.12.002 20045007

[B23] Li S. Dang Y. Zhou X. Huang B. Huang X. Zhang Z. (2015). Formononetin Promotes Angiogenesis through the Estrogen Receptor Alpha-Enhanced ROCK Pathway. Sci. Rep. 5, 16815. 10.1038/srep16815 26568398 PMC4645220

[B24] Li S. Guan J.-L. Chien S. (2005). Biochemistry and Biomechanics of Cell Motility. Annu. Rev. Biomed. Eng. 7, 105–150. 10.1146/annurev.bioeng.7.060804.100340 16004568

[B25] Limbourg A. Korff T. Napp L. C. Schaper W. Drexler H. Limbourg F. P. (2009). Evaluation of Postnatal Arteriogenesis and Angiogenesis in a Mouse Model of Hind-Limb Ischemia. Nat. Protoc. 4 (12), 1737–1748. 10.1038/nprot.2009.185 19893509

[B26] Liu C.-H. Chen M.-F. Tseng T.-L. Chen L.-G. Kuo J.-S. Lee T. J.-F. (2012). Oroxylin A, but Not Vasopressin, Ameliorates Cardiac Dysfunction of Endotoxemic Rats. Evidence-Based Complement. Altern. Med. 2012, 1–12. 10.1155/2012/408187 PMC3489109 23193421

[B27] Liu J. Wada Y. Katsura M. Tozawa H. Erwin N. Kapron C. M. (2018). Rho-Associated Coiled-Coil Kinase (ROCK) in Molecular Regulation of Angiogenesis. Theranostics 8, 6053–6069. 10.7150/thno.30305 30613282 PMC6299434

[B28] McDermott M. M. Kibbe M. R. (2017). Improving Lower Extremity Functioning in Peripheral Artery Disease. JAMA 317 (7), 689–690. 10.1001/jama.2016.20673 28241363

[B29] Meins M. Henderson D. J. Bhattacharya S. S. Sowden J. C. (2000). Characterization of the Human TBX20 Gene, a New Member of the T-Box Gene Family Closely Related to the Drosophila H15 Gene. Genomics 67, 317–332. 10.1006/geno.2000.6249 10936053

[B30] Meng S. Gu Q. Yang X. Lv J. Owusu I. Matrone G. (2018). TBX20 Regulates Angiogenesis through the Prokineticin 2-Prokineticin Receptor 1 Pathway. Circulation 138, 913–928. 10.1161/circulationaha.118.033939 29545372 PMC6139092

[B31] Ministro A. de Oliveira P. Nunes R. J. Dos Santos Rocha A. Correia A. Carvalho T. (2017). Low-dose Ionizing Radiation Induces Therapeutic Neovascularization in a Pre-clinical Model of Hindlimb Ischemia. Cardiovasc. Res. 113 (7), 783–794. 10.1093/cvr/cvx065 28444128

[B32] Minoshima A. Kabara M. Matsuki M. Yoshida Y. Kano K. Tomita Y. (2018). Pericyte-specific Ninjurin1 Deletion Attenuates Vessel Maturation and Blood Flow Recovery in Hind Limb Ischemia. Atvb 38 (10), 2358–2370. 10.1161/ATVBAHA.118.311375 PMC6166707 30354207

[B33] Mittermayr R. Slezak P. Haffner N. Smolen D. Hartinger J. Hofmann A. (2016). Controlled Release of Fibrin Matrix-Conjugated Platelet Derived Growth Factor Improves Ischemic Tissue Regeneration by Functional Angiogenesis. Acta Biomater. 29, 11–20. 10.1016/j.actbio.2015.10.028 26497625

[B34] Murakami M. Nguyen L. T. Zhang Z. W. Moodie K. L. Carmeliet P. Stan R. V. (2008). The FGF System Has a Key Role in Regulating Vascular Integrity. J. Clin. Invest. 118, 3355–3366. 10.1172/jci35298 18776942 PMC2528913

[B35] Potente M. Gerhardt H. Carmeliet P. (2011). Basic and Therapeutic Aspects of Angiogenesis. Cell 146, 873–887. 10.1016/j.cell.2011.08.039 21925313

[B36] Qin Y. Wang J.-b. Kong W.-j. Zhao Y.-l. Yang H.-y. Dai C.-m. (2011). The Diarrhoeogenic and Antidiarrhoeal Bidirectional Effects of Rhubarb and its Potential Mechanism. J. Ethnopharmacology 133, 1096–1102. 10.1016/j.jep.2010.11.041 21112382

[B37] Rajasagi N. K. Bhela S. Varanasi S. K. Rouse B. T. (2017). Frontline Science: Aspirin-Triggered Resolvin D1 Controls Herpes Simplex Virus-Induced Corneal Immunopathology. J. Leukoc. Biol. 102 (5), 1159–1171. 10.1189/jlb.3hi1216-511rr 28584076 PMC5636045

[B38] Ren G. Chen H. Zhang M. Yang N. Yang H. Xu C. (2020). Pharmacokinetics, Tissue Distribution and Excretion Study of Oroxylin A, Oroxylin A 7-O-Glucuronide and Oroxylin A Sodium Sulfonate in Rats after Administration of Oroxylin A. Fitoterapia 142, 104480. 10.1016/j.fitote.2020.104480 31927013

[B39] Roncalli J. Tongers J. Renault M.-A. Losordo D. W. (2008). Biological Approaches to Ischemic Tissue Repair: Gene- and Cell-Based Strategies. Expert Rev. Cardiovasc. Ther. 6 (5), 653–668. 10.1586/14779072.6.5.653 18510483

[B40] Saleh A. Stathopoulou M. G. Dadé S. Ndiaye N. C. Azimi-Nezhad M. Murray H. (2015). Angiogenesis Related Genes NOS3, CD14, MMP3 and IL4R Are Associated to VEGF Gene Expression and Circulating Levels in Healthy Adults. BMC Med. Genet. 16, 90. 10.1186/s12881-015-0234-6 26437765 PMC4594922

[B41] Scagliotti G. V. Gaafar R. Nowak A. K. Nakano T. van Meerbeeck J. Popat S. (2019). Nintedanib in Combination with Pemetrexed and Cisplatin for Chemotherapy-Naive Patients with Advanced Malignant Pleural Mesothelioma (LUME-Meso): a Double-Blind, Randomised, Placebo-Controlled Phase 3 Trial. Lancet Respir. Med. 7 (7), 569–580. 10.1016/S2213-2600(19)30139-0 31103412

[B42] Skovseth D. K. Veuger M. J. T. Sorensen D. R. De Angelis P. M. Haraldsen G. (2005). Endostatin Dramatically Inhibits Endothelial Cell Migration, Vascular Morphogenesis, and Perivascular Cell Recruitment *In Vivo* . Blood 105, 1044–1051. 10.1182/blood-2004-03-1164 15466935

[B43] Song X. Chen Y. Sun Y. Lin B. Qin Y. Hui H. (2012). Oroxylin A, a Classical Natural Product, Shows a Novel Inhibitory Effect on Angiogenesis Induced by Lipopolysaccharide. Pharmacol. Rep. 64 (5), 1189–1199. 10.1016/s1734-1140(12)70915-5 23238475

[B44] Sun H.-J. Cai W.-W. Gong L.-L. Wang X. Zhu X.-X. Wan M.-Y. (2017). FGF-2-mediated FGFR1 Signaling in Human Microvascular Endothelial Cells Is Activated by Vaccarin to Promote Angiogenesis. Biomed. Pharmacother 95, 144–152. 10.1016/j.biopha.2017.08.059 28841454

[B45] Suzuki J.-i. Shimamura M. Suda H. Wakayama K. Kumagai H. Ikeda Y. (2016). Current Therapies and Investigational Drugs for Peripheral Arterial Disease. Hypertens. Res. 39, 183–191. 10.1038/hr.2015.134 26631852

[B46] Tseng T.-L. Chen M.-F. Liu C.-H. Pang C.-Y. Hsu Y.-H. Lee T. J. F. (2016). Induction of Endothelium-dependent Constriction of Mesenteric Arteries in Endotoxemic Hypotensive Shock. Br. J. Pharmacol. 173 (7), 1179–1195. 10.1111/bph.13415 26694894 PMC5341340

[B47] Tseng T.-L. Chen M.-F. Tsai M.-J. Hsu Y.-H. Chen C.-P. Lee T. J. F. (2012). Oroxylin-A Rescues LPS-Induced Acute Lung Injury via Regulation of NF-κB Signaling Pathway in Rodents. PLoS One 7 (10), e47403. 10.1371/journal.pone.0047403 23071799 PMC3468516

[B48] Van Nieuw Amerongen G. P. Koolwijk P. Versteilen A. Van Hinsbergh V. W. M. (2003). Involvement of RhoA/Rho Kinase Signaling in VEGF-Induced Endothelial Cell Migration and Angiogenesis *In Vitro* . Atvb 23 (2), 211–217. 10.1161/01.ATV.0000054198.68894.88 12588761

[B49] Wang H. Guo Y. Zhao X. Li H. Fan G. Mao H. (2013). An Estrogen Receptor Dependent Mechanism of Oroxylin A in the Repression of Inflammatory Response. PLoS One 8, e69555. 10.1371/journal.pone.0069555 23922737 PMC3726624

[B50] Warner H. Wilson B. J. Caswell P. T. (2019). Control of Adhesion and Protrusion in Cell Migration by Rho GTPases. Curr. Opin. Cell Biol. 56, 64–70. 10.1016/j.ceb.2018.09.003 30292078 PMC6368645

[B51] Yano T. Yamazaki Y. Adachi M. Okawa K. Fort P. Uji M. (2011). Tara Up-Regulates E-Cadherin Transcription by Binding to the Trio RhoGEF and Inhibiting Rac Signaling. J. Cell Biol. 193, 319–332. 10.1083/jcb.201009100 21482718 PMC3080255

[B52] Yin Y. Duan J. Guo C. Wei G. Wang Y. Guan Y. (2017). Danshensu Accelerates Angiogenesis after Myocardial Infarction in Rats and Promotes the Functions of Endothelial Progenitor Cells through SDF-1α/CXCR4 axis. Eur. J. Pharmacol. 814, 274–282. 10.1016/j.ejphar.2017.08.035 28864209

[B53] Zhang C. Bian M. Chen X. Jin H. Zhao S. Yang X. (2018). Oroxylin A Prevents Angiogenesis of LSECs in Liver Fibrosis via Inhibition of YAP/HIF‐1α Signaling. J. Cell. Biochem. 119 (2), 2258–2268. 10.1002/jcb.26388 28857294

[B54] Zhang L.-j. Chen L. Lu Y. Wu J.-m. Xu B. Sun Z.-g. (2010). Danshensu Has Anti-tumor Activity in B16F10 Melanoma by Inhibiting Angiogenesis and Tumor Cell Invasion. Eur. J. Pharmacol. 643 (2-3), 195–201. 10.1016/j.ejphar.2010.06.045 20621088

[B55] Zhang M. Gao F. Teng F. Zhang C. (2014). Tetramethylpyrazine Promotes the Proliferation and Migration of Brain Endothelial Cells. Mol. Med. Rep. 10, 29–32. 10.3892/mmr.2014.2169 24789060 PMC4068727

[B56] Zhang M. J. Sansbury B. E. Hellmann J. Baker J. F. Guo L. Parmer C. M. (2016). Resolvin D2 Enhances Postischemic Revascularization while Resolving Inflammation. Circulation 134, 666–680. 10.1161/circulationaha.116.021894 27507404 PMC5214591

[B57] Zhao K. Li X. Lin B. Yang D. Zhou Y. Li Z. (2018). Oroxyloside Inhibits Angiogenesis through Suppressing Internalization of VEGFR2/Flk-1 in Endothelial Cells. J. Cell. Physiol. 233 (4), 3454–3464. 10.1002/jcp.26198 28926106

