# Supplementary figures and images for: Oroxylin a Attenuates Limb Ischemia by Promoting Angiogenesis via Modulation of Endothelial Cell Migration
Source: Front Pharmacol. 2021 Jul 30;12:705617. doi: 10.3389/fphar.2021.705617 (PMC8370028; doi:10.3389/fphar.2021.705617)

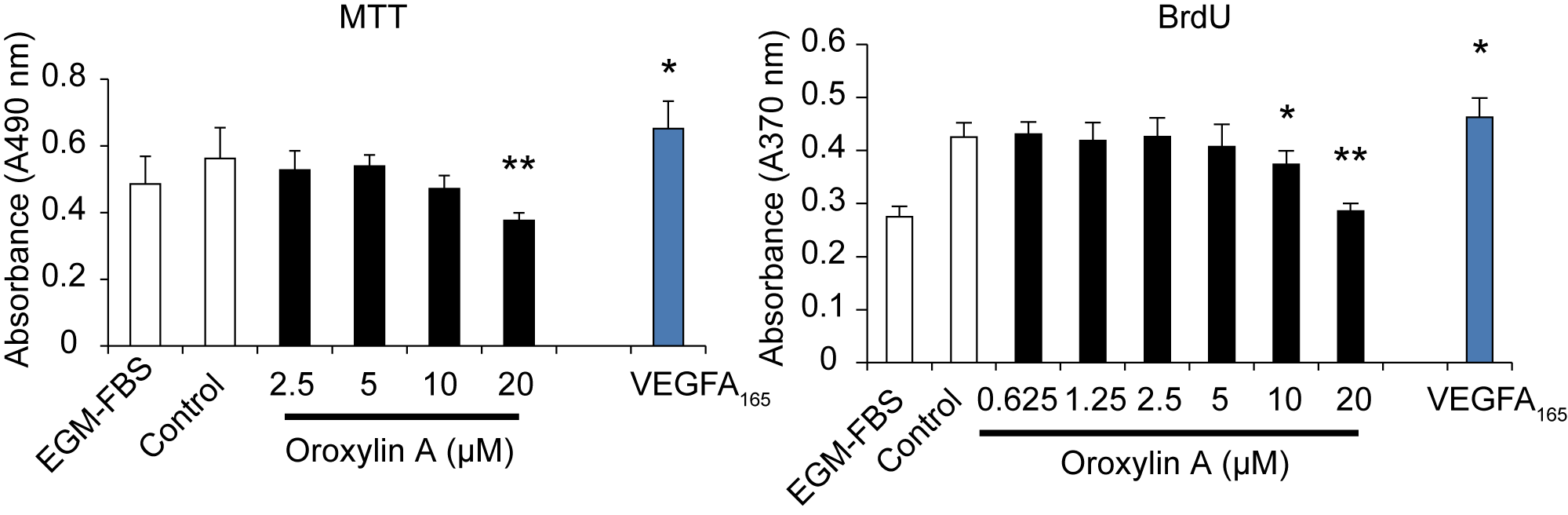

Supplement: Supplementary file 1 [file image3.tif]

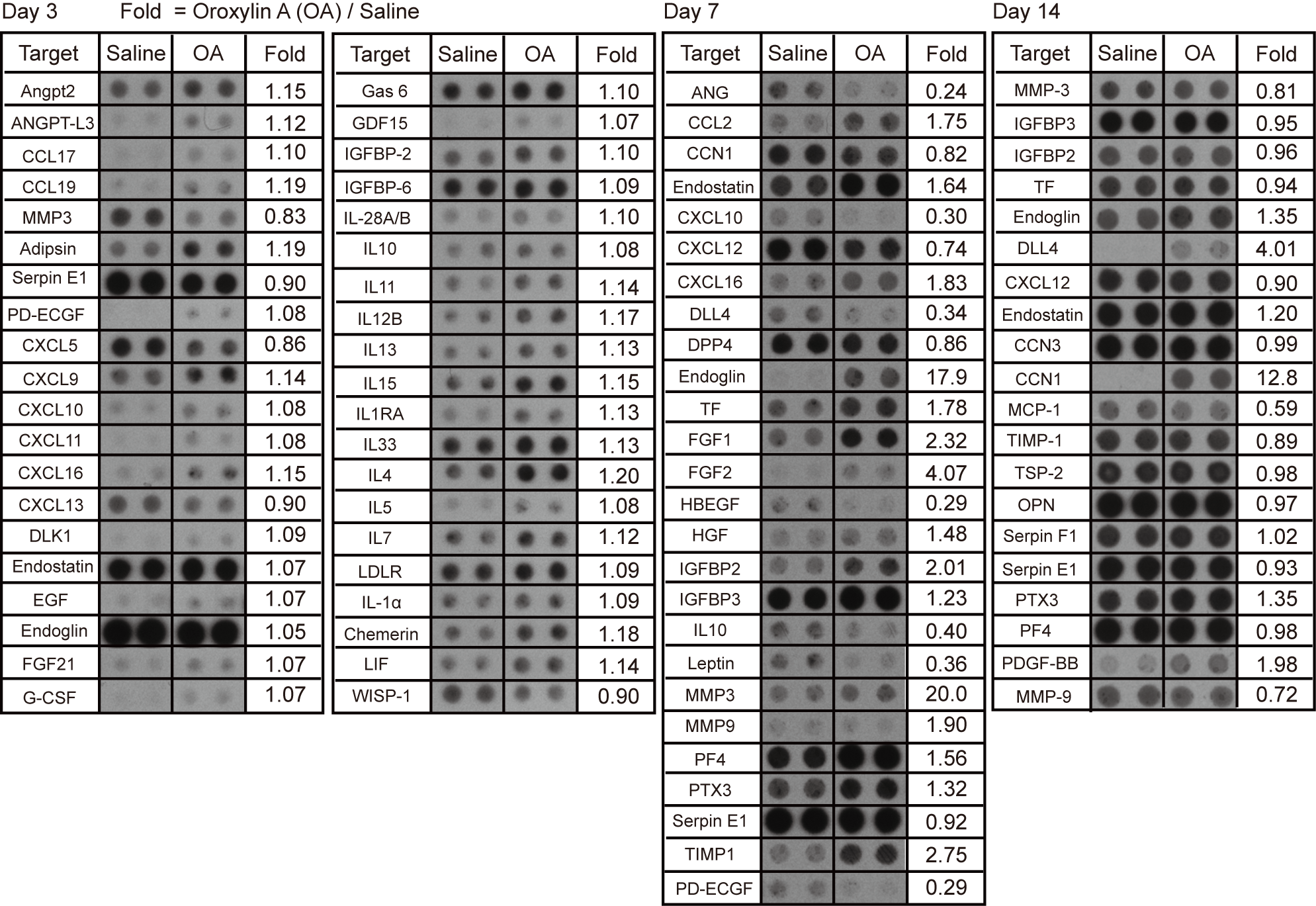

Supplement: Supplementary file 2 [file image2.tif]

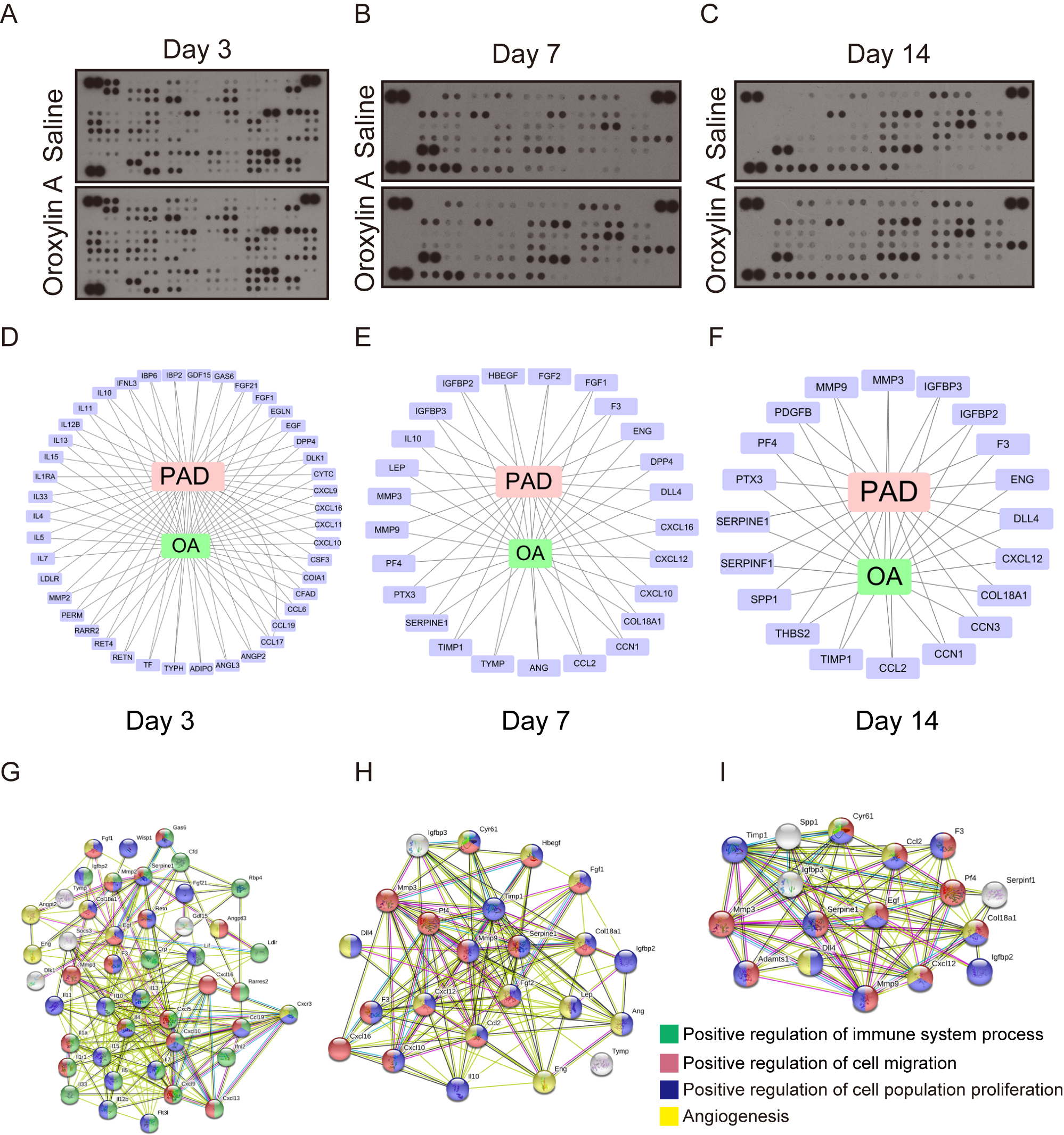

Supplement: Supplementary file 3 [file image1.tif]
