# Supplementary material for: Oroxylin a Attenuates Limb Ischemia by Promoting Angiogenesis via Modulation of Endothelial Cell Migration
Source: Front Pharmacol. 2021 Jul 30;12:705617. doi: 10.3389/fphar.2021.705617 (PMC8370028; doi:10.3389/fphar.2021.705617)
Supplement: Supplementary file 4 [file datasheet1.pdf]

Fig.2 Western blotting

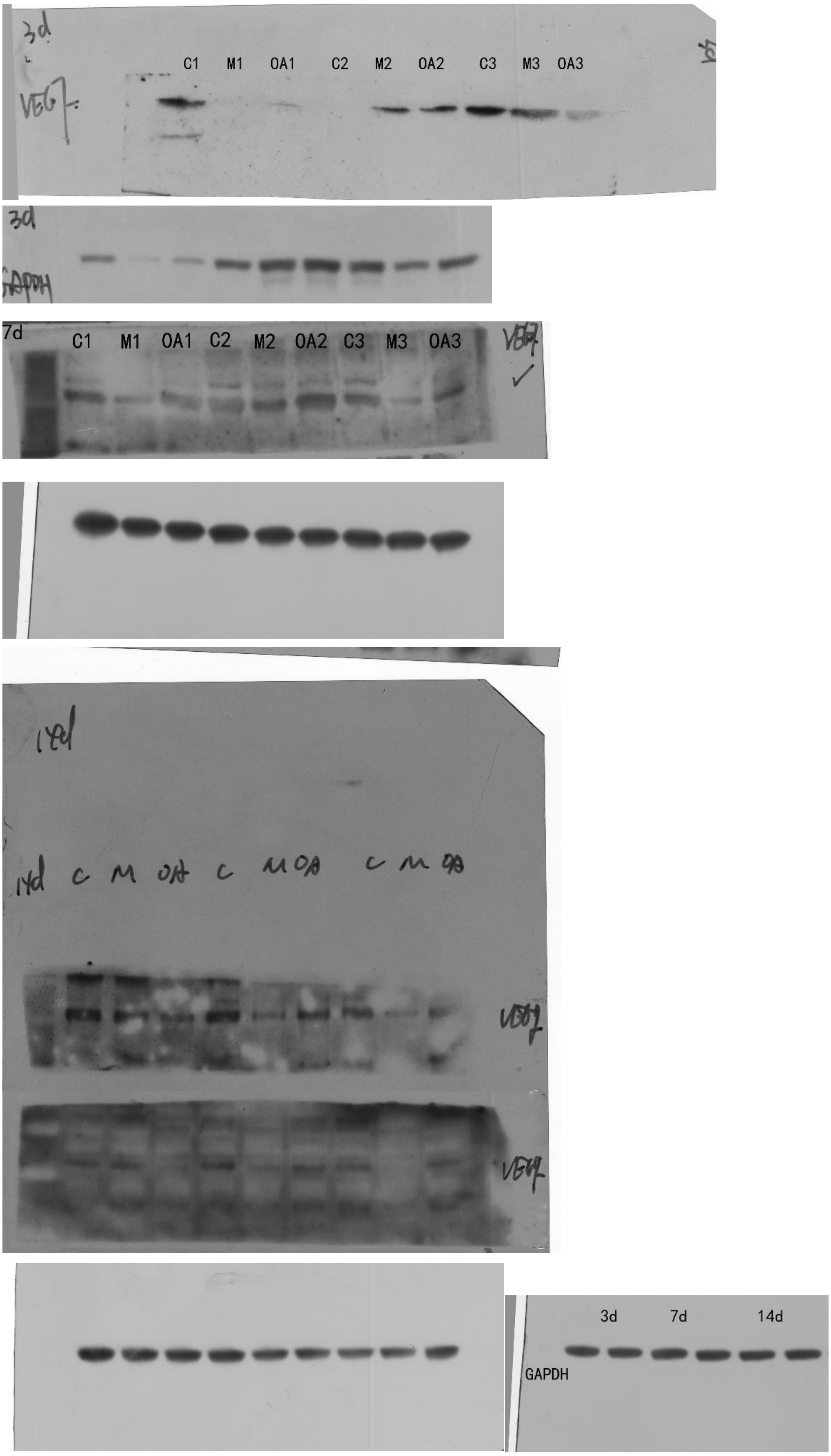

Fig 4

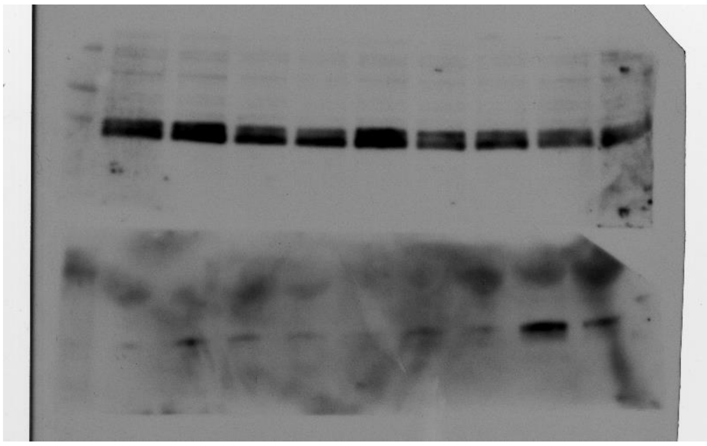

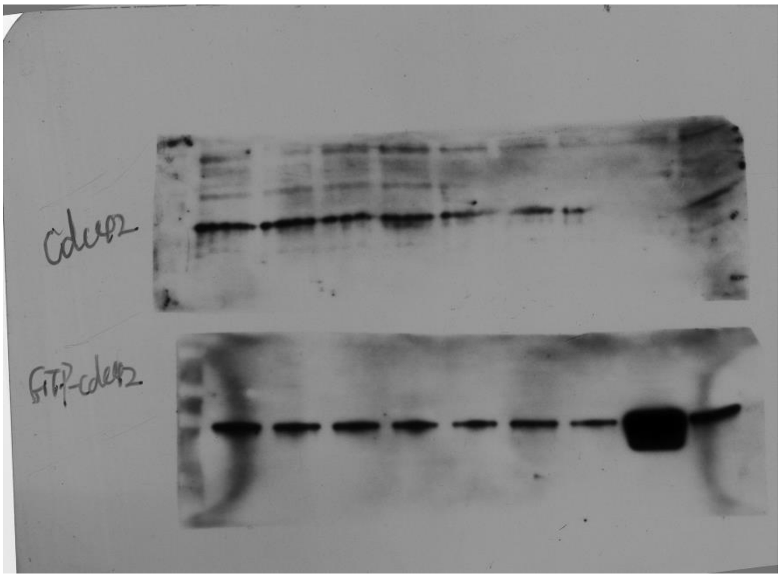

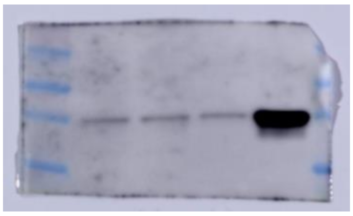

GTP-cdc42

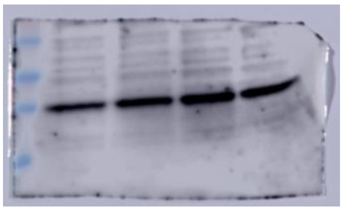

cdc42 total

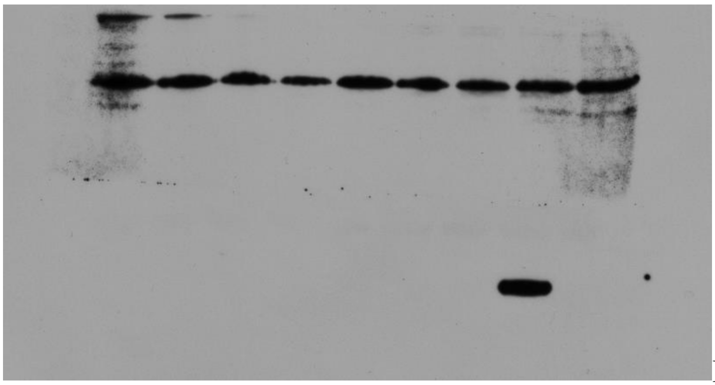

Rac-1 total GTP-Rac-1

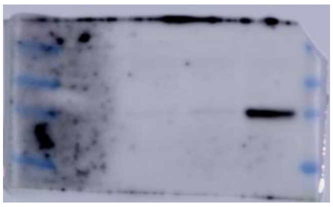
GTP-Rac-1

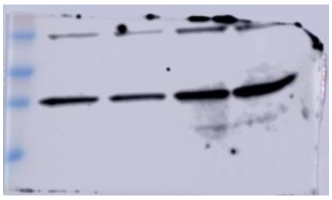
Rac-1 total

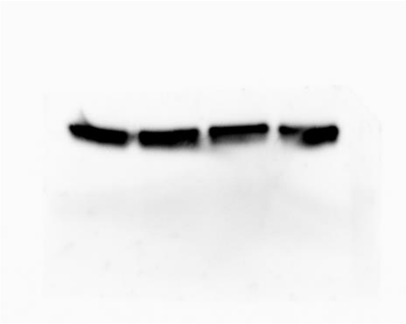
cofilin

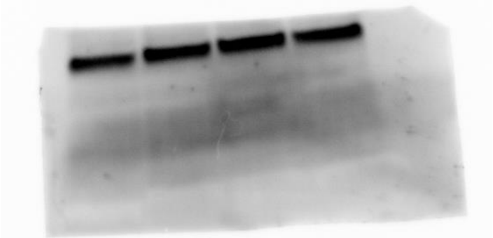
p-cofilin

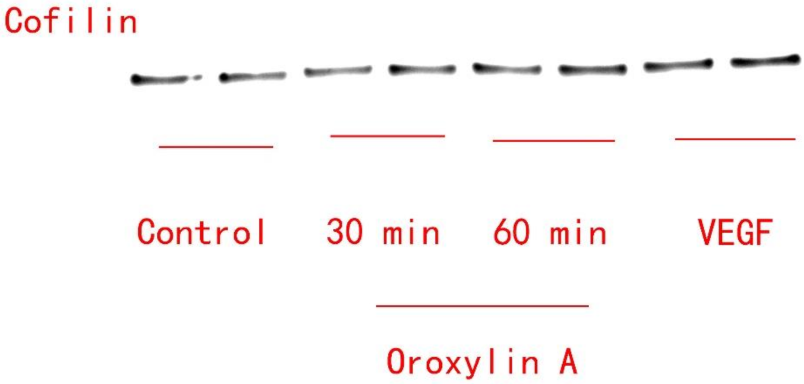

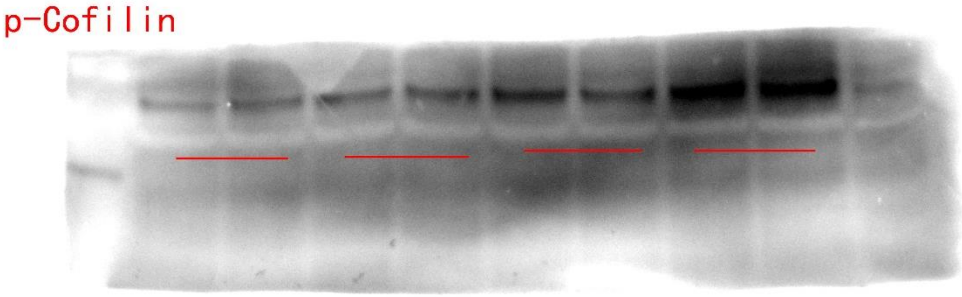

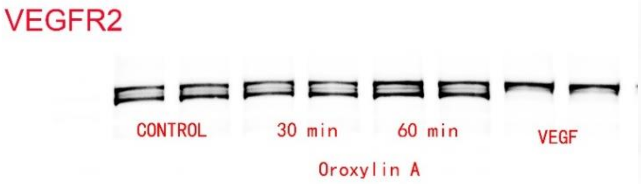

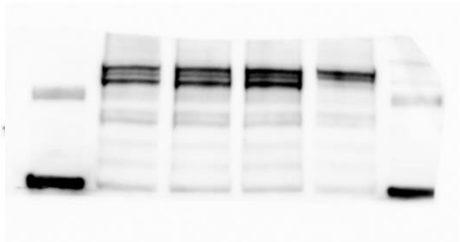

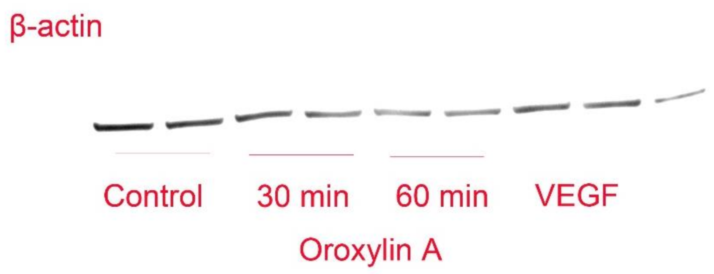

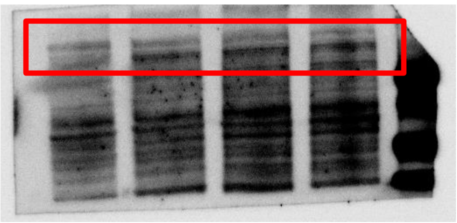
p-vegfr2

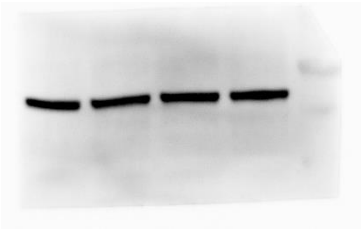
β-actin

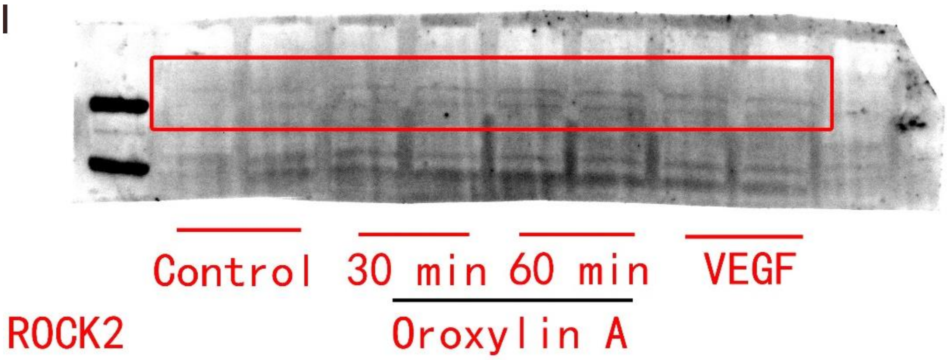

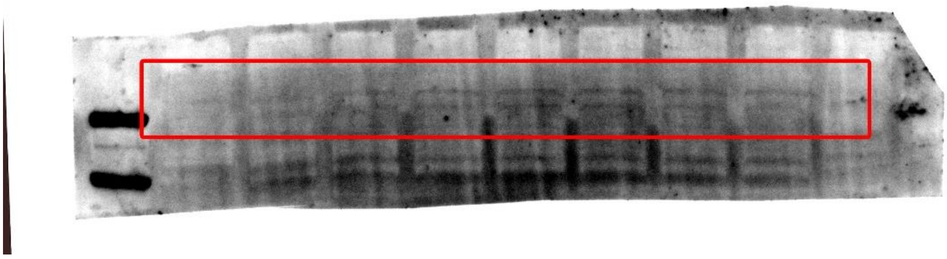

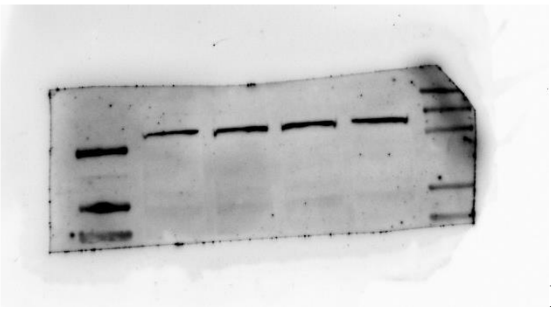
ROCK2(ROCKII)

Fig 5

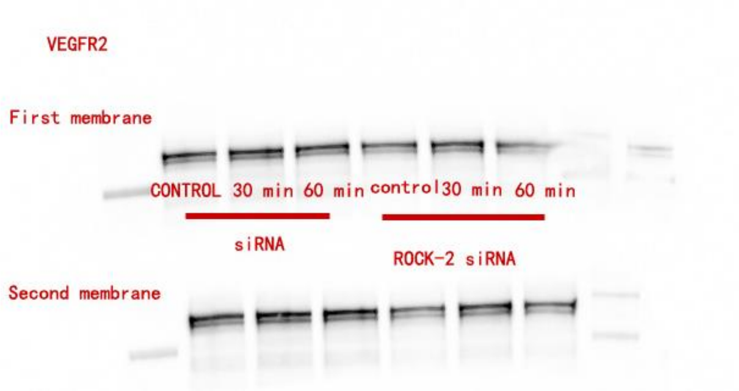

β-actin

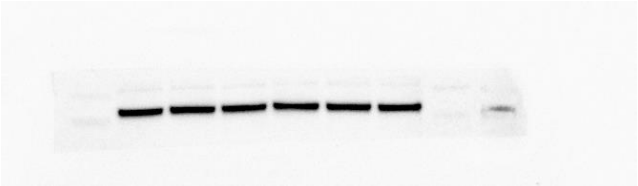

ROCK2

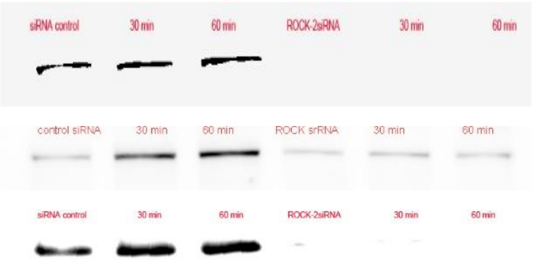

β-actin

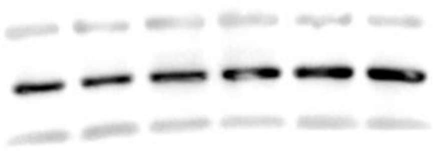

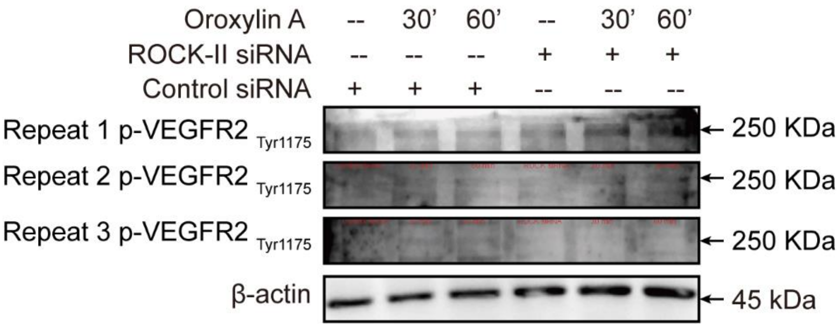

Fig 6

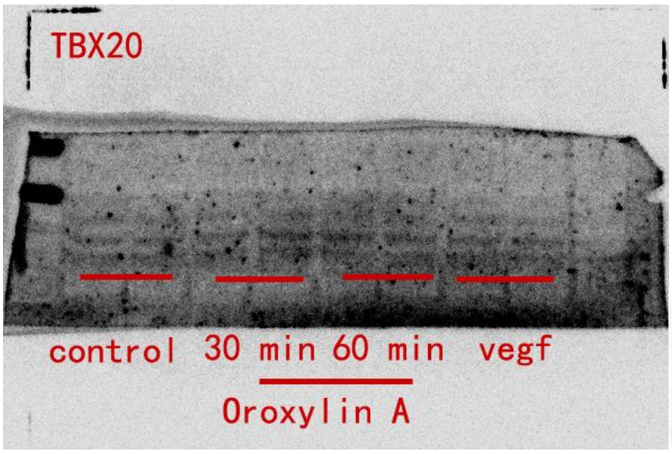

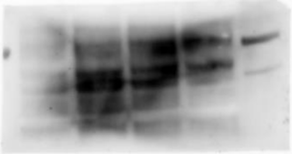

TBX20

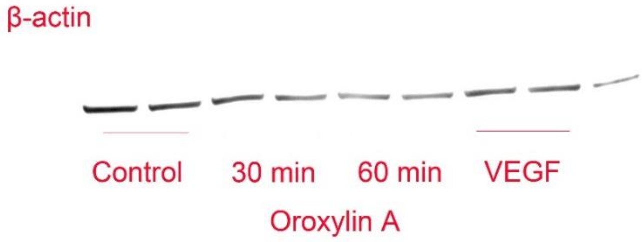

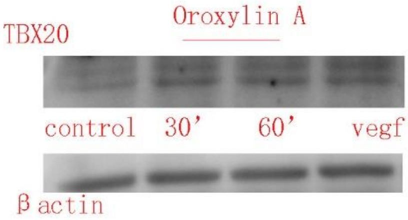

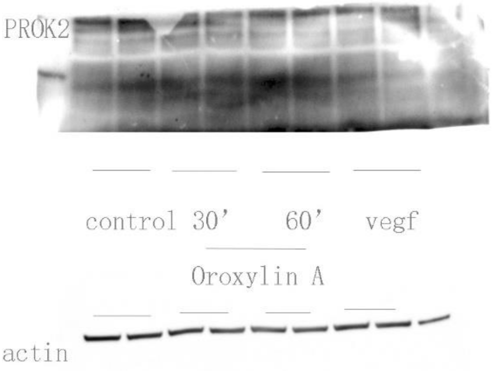

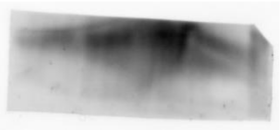

PROK2

β-actin

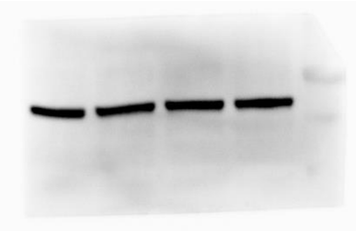

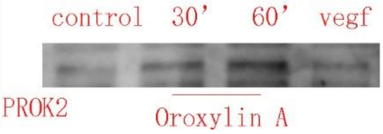

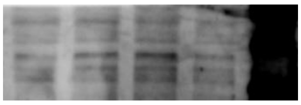

supply fig

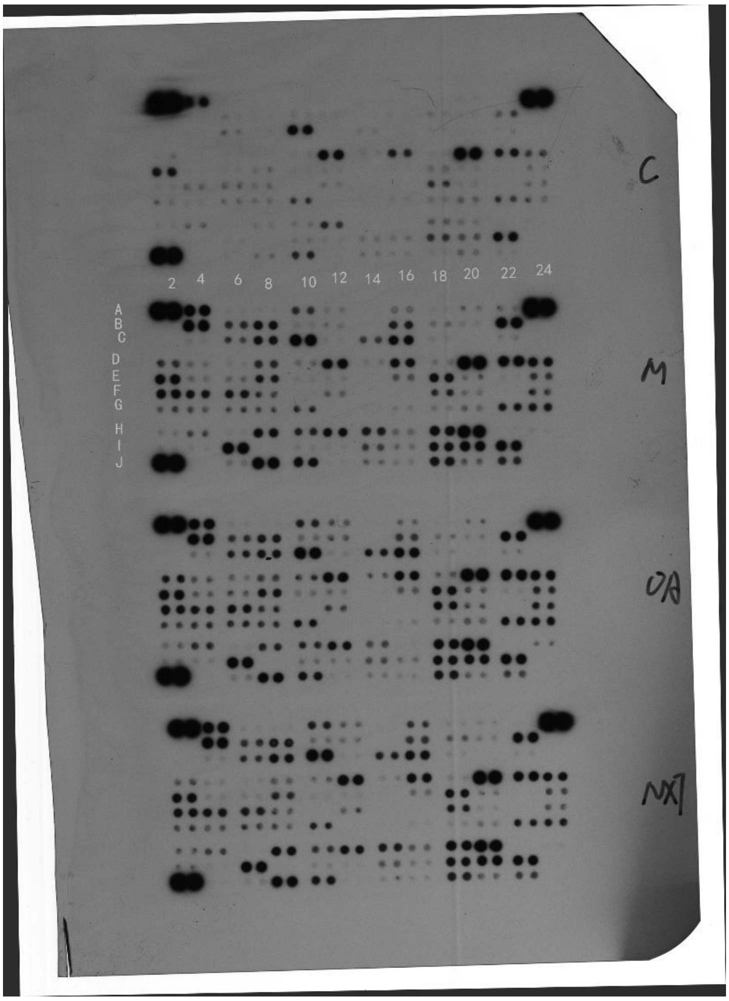

3d

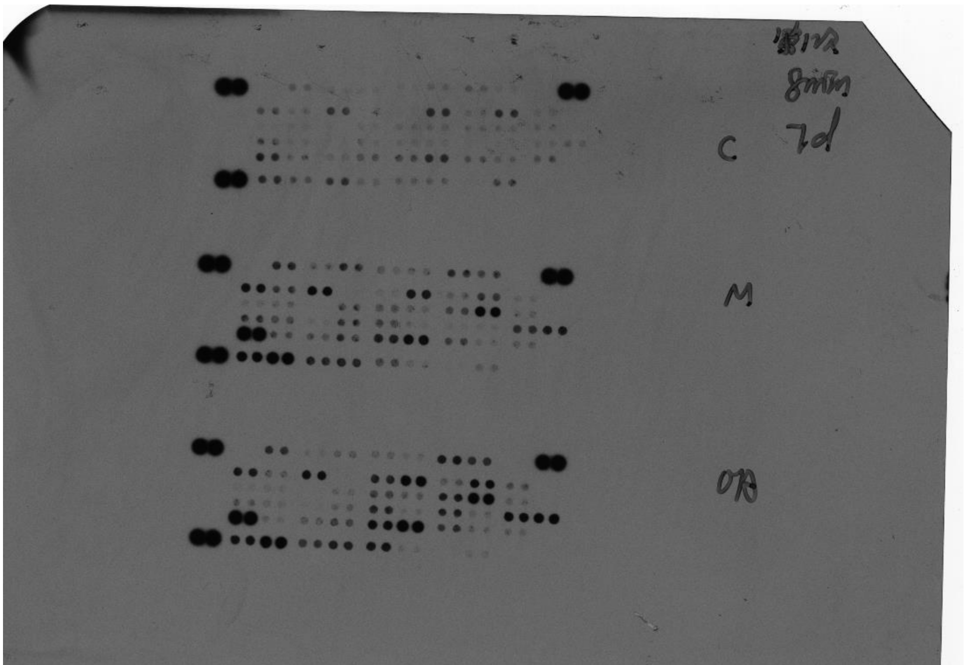

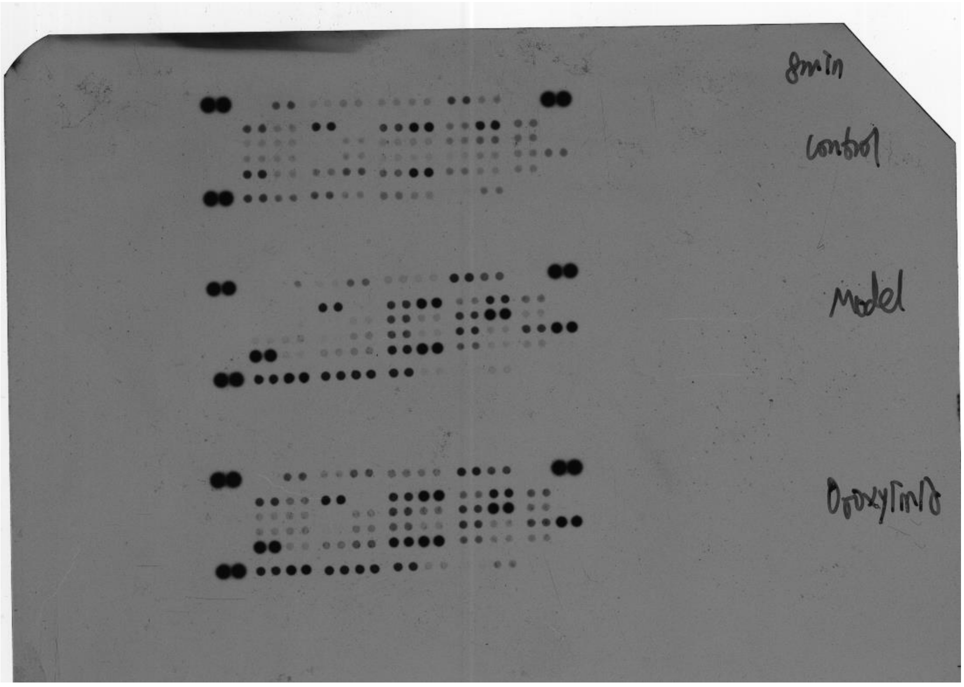

14d 8min
